# Whole genome variation in 27 Mexican indigenous populations, demographic and biomedical insights

**DOI:** 10.1371/journal.pone.0249773

**Published:** 2021-04-08

**Authors:** Israel Aguilar-Ordoñez, Fernando Pérez-Villatoro, Humberto García-Ortiz, Francisco Barajas-Olmos, Judith Ballesteros-Villascán, Ram González-Buenfil, Cristobal Fresno, Alejandro Garcíarrubio, Juan Carlos Fernández-López, Hugo Tovar, Enrique Hernández-Lemus, Lorena Orozco, Xavier Soberón, Enrique Morett

**Affiliations:** 1 Instituto de Biotecnología, Universidad Nacional Autónoma de México (UNAM), Cuernavaca, Morelos, México; 2 Instituto Nacional de Medicina Genómica (INMEGEN), Mexico City, México; 3 Winter Genomics, Mexico City, México; 4 Benemérita Universidad Autónoma de Puebla (BUAP), Puebla de Zaragoza, Puebla, México; Case Western Reserve University, UNITED STATES

## Abstract

There has been limited study of Native American whole genome diversity to date, which impairs effective implementation of personalized medicine and a detailed description of its demographic history. Here we report high coverage whole genome sequencing of 76 unrelated individuals, from 27 indigenous groups across Mexico, with more than 97% average Native American ancestry. On average, each individual has 3.26 million Single Nucleotide Variants and short indels, that together comprise a catalog of 9,737,152 variants, 44,118 of which are novel. We report 497 common Single Nucleotide Variants (with allele frequency > 5%) mapped to drug responses and 316,577 in enhancer or promoter elements; interestingly we found some of these enhancer variants in PPARG, a nuclear receptor involved in highly prevalent health problems in Mexican population, such as obesity, diabetes, and insulin resistance. By detecting signals of positive selection we report 24 enriched key pathways under selection, most of them related to immune mechanisms. No missense variants in ACE2, the receptor responsible for the entry of the SARS CoV-2 virus, were found in any individual. Population genomics and phylogenetic analyses demonstrated stratification in a Northern-Central-Southern axis, with major substructure in the Central region. The Seri, a northern group with the most genetic divergence in our study, showed a distinctive genomic context with the most novel variants, and the most population specific genotypes. Genome-wide analysis showed that the average haplotype blocks are longer in Native Mexicans than in other world populations. With this dataset we describe previously undetected population level variation in Native Mexicans, helping to reduce the gap in genomic data representation of such groups.

## Introduction

The study of the biology and evolutionary history of our species, as well as effective implementation of genomic medicine, require a broad understanding of the human genomic variation across the world. While some genomic variation is shared by populations from all continents, which is described as common or ancient variation, other polymorphisms of more recent origin are limited to specific regions, which are responsible for population specific phenotypic traits and health risks [1, 2]. Native Americans (NatAm) have been through a deep and rapid population transition in the recent history of our species.Most of the highly diverse contemporary NatAm populations descend from a small group of inhabitants of Siberia [3] and some other streams of ancestral groups [4–7]. Once the last glaciation ended, about 15,000 years ago, ancient NatAm readily moved along the whole continent and populated a wide array of ecosystems [8], ranging from coastal areas, to deserts, mountain ranges and tropical forests. This fast expansion along a 16 thousand kilometers North-South axis into an uninhabited continent, is unparalleled in the history of human groups [3, 5].The peopling of the continent was complex and there is evidence of back-migrations along other interesting population movements [5]. Genetic changes that took place in these new ecosystems were certainly swift and with far reaching consequences, leaving marks in the genomes of present day NatAm [9].

The region that currently comprises Mexico was settled by small populations that rapidly grew after agriculture was implemented in Central Mexico [10]. Native Mexicans (NM) that settled in the North continued living mainly as hunter gatherers, a lifestyle not compatible with large populations [11, 12]. The arrival of the Spanish conquerors in the XV century dramatically changed the population landscape of the whole American continent [13], such that contemporary people from Mexico are a heterogeneous admixed group of populations [14], composed by the genomic heritage from different regional NM backgrounds, Europeans, and, to a lesser extent, West African and Asian populations [15]. However, millions of individuals still have a very marked indigenous identity with a broad cultural heterogeneity along the country. This cultural heterogeneity is paralleled by a genetic divergence revealed by genomic analyses; regionally, within a single continent, NM show a range of genomic differentiation comparable in scope to the differentiation between the European and East Asian continental populations [14, 16]. Some of the NM groups, like the Seri, a northern population, have a particular history as hunter-gatherers, which combined with a cultural context favoring isolation, has probably shaped an interesting genomic context yet deeply unexplored [12].

The genetic structure of NM populations has previously been analyzed using SNP-array and exome sequencing technologies [14, 15, 17]. However, to get a complete and genome-wide picture of variation, it is essential to expand its study by Whole Genome Sequencing (WGS). International WGS efforts have studied people closely related to the current Mexican population [4, 18, 19], but none of those projects focused on the particularities of NM populations. To date, only 107 whole genome sequences of Mexican individuals with highly admixed ancestries [1], and 54 individuals with a high degree of NM ancestry have been studied and are publicly available [16, 18, 19]. Here we extended the number of studied NM genomes with the high coverage WGS of 76 unrelated NM individuals from 27 indigenous groups. Our study contributes to a better definition of the genomic diversity of NM and improves the understanding of their population structure, relatedness, and demographic history.

## Results and discussion

### NM variation catalogue

We sequenced whole genomes of 94 individuals 78 years of age or older from 32 indigenous groups across Mexico using Illumina HiSeq X Ten. It is worth noting that ethnic groups are identified mainly by their language, and that those languages are also grouped into linguistic families. Besides, some languages are spoken in vast regions of Mexico, so a geographic subdivision is warranted; for instance, Nahua (a language group) is subdivided into Morelos, San Luis Potosí, and Puebla (which are 3 of the 32 geographic states of Mexico) (S1 Table in S2 Material).

**Fig 1:**
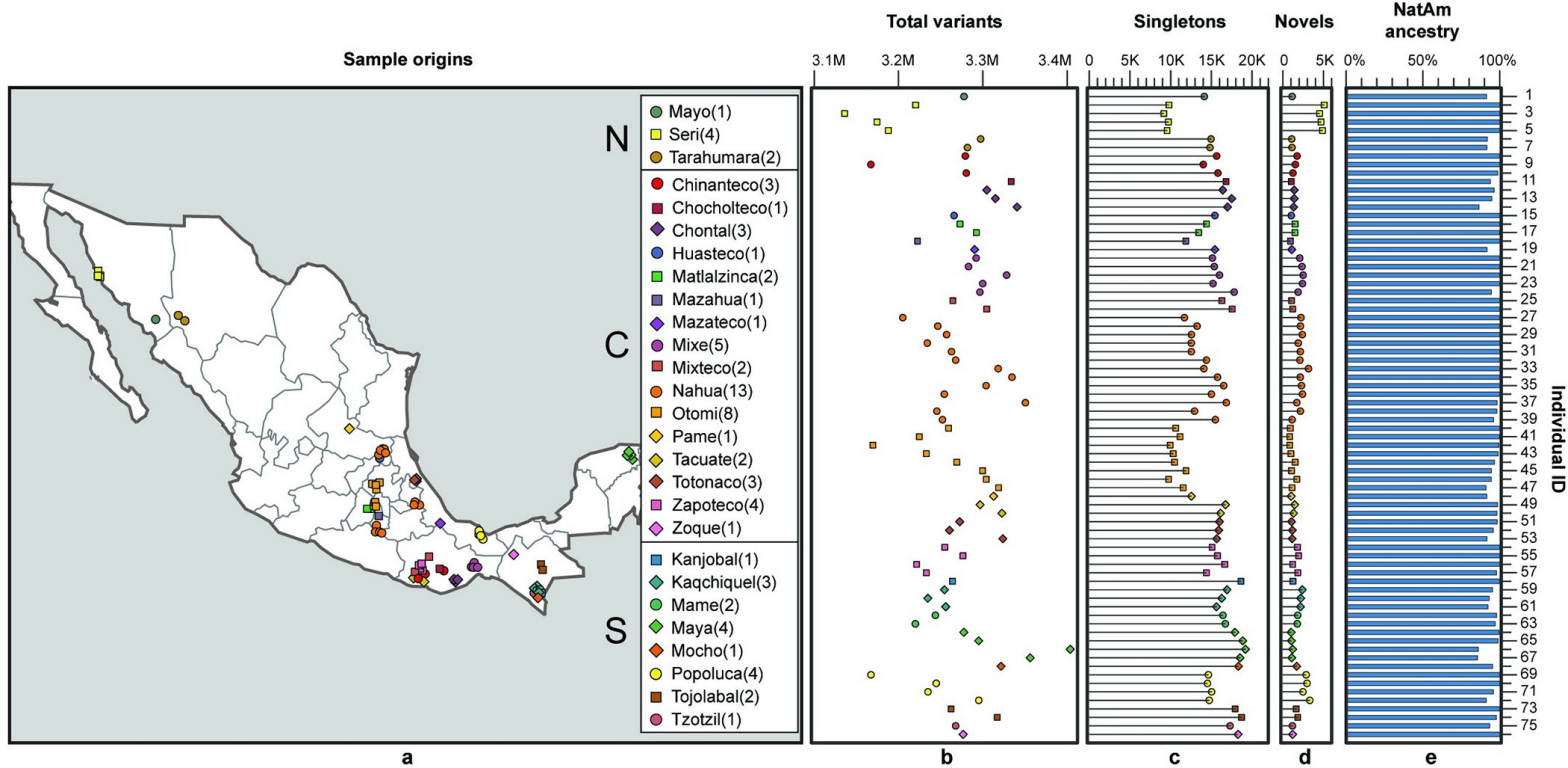
NM sampling. (a) Each point represents the approximate geographic origin of the 76 individuals, and the number in the legend indicates the number of samples per indigenous group. Legend separates individuals from Northern (N), Central (C) and Southern (S) regions. For the exact GPS coordinates see S1 Table in S2 Material. (b) Total variants (M = millions) for each of the 76 individuals; the y axis enumerates the sampled individuals and is shared with panels c, d, and e; shape and color of the points correspond to the indigenous groups in the map. (c) Number of singletons (K = thousands) for each sample inferred from worldwide comparison with gnomAD and the 1000 Genomes Project. (d) Number of novel variants (K = thousands) not registered in dbSNP b152. (e) Percentage of Native American ancestry.

After filtering out individuals related up to a second degree, as well as those with less than 85% global NatAm ancestry, estimated by ADMIXTURE [20], we continued the study with 76 samples corresponding to 40 females and 36 males, from 27 indigenous groups (Fig 1A; S1 Table in S2 Material); European ancestry in dropped samples ranged from 10% to 58%, a reflection of the non-uniformity of admixed ancestry in the country (S1 Note in S1 Material). The average NatAm ancestry of the 76 samples included in our study was 97.22% (S2 Table in S2 Material). Sequencing reads were aligned using the SNAP aligner [21]. Approximately 95% of the GRCh38 genome was covered with a mean depth of 24X (S3 Table in S2 Material). This represents 100.6 Mbp previously uncovered by gnomAD 2.1, the largest population sequencing repository [22] (S4 Table in S2 Material). The difference in coverage is due to the fact that gnomAD 2.1 used the GRCh37 genome as reference, and that it does not report chrY data. As such, our project describes a substantial proportion of novel population level variation in NM.

**Fig 2:**
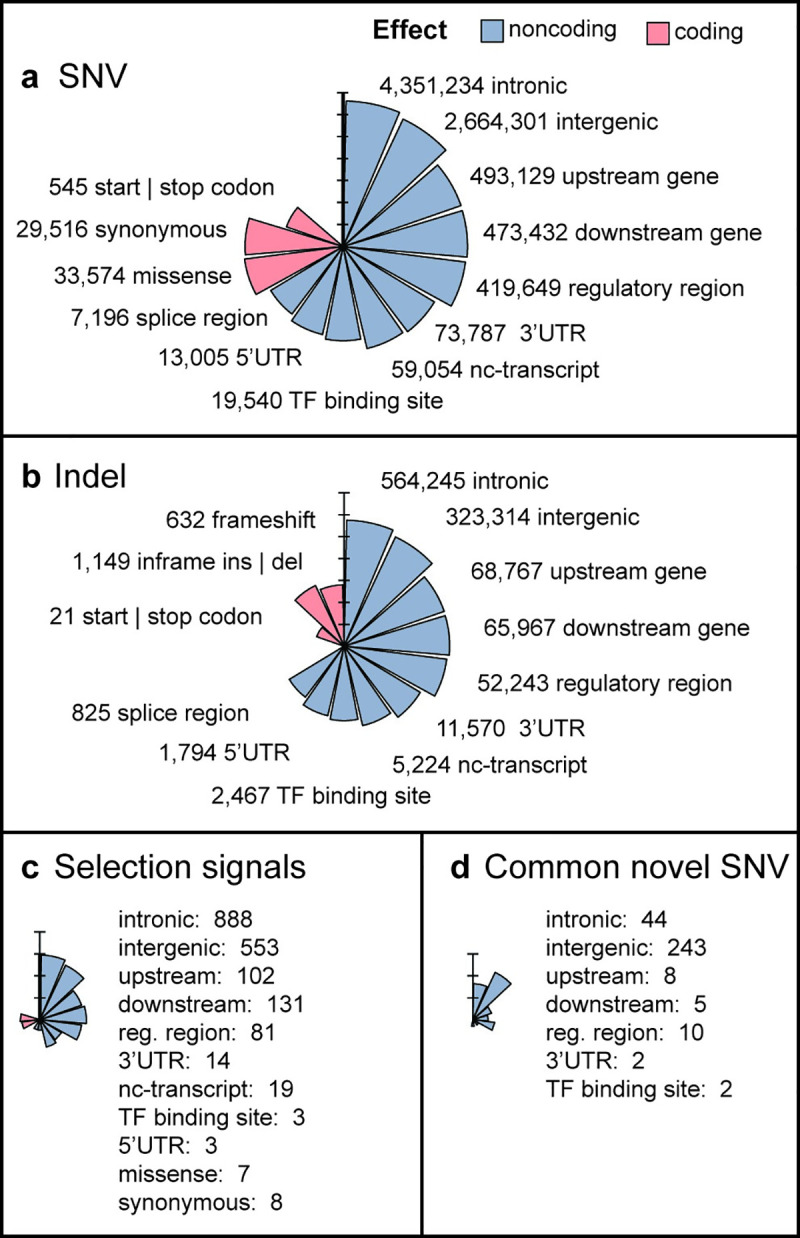
Summary of variant effect annotations in the NM catalog. All plots depict log10 number of variants. The color legend is shared between panels. (a) Consequences from the full set of SNVs. (b) Consequences from the full set of indels. (c) Consequences in natural selection signals. (d) Consequences in novel SNVs found at an allele frequency > 5%. nc-transcript = noncoding transcript.

Short variant calling was performed following the GATK best practices protocol [23]; variants from each individual were integrated into a Joint Population Variant Set (JPVS) including only sites with call rate > 98.5%. This dataset comprises a catalogue of 8,638,130 SNVs and 1,099,022 short indels. The JPVS includes 44,118 novel variants, not registered in dbSNP b152 [24]. Variant calling accuracy was evaluated by comparing SNP-array data of the same samples (6.0 SNP array, Affymetrix), with an average of > 99% concordance (S1 Table in S2 Material). Each individual carried on average 3,262,160 variants (Fig 1B; S1 Table in S2 Material), a number within the range (~ 2.7 to 3.7 millions) of other regional projects [25–28]. Approximately 0.7% of SNVs and 0.2% of indels affected coding regions (Fig 2; S5 Table in S2 Material). Transition to transversion mean ratio (Ts/Tvs) was 2.127. The Seri group showed the lowest number of worldwide singletons, and the highest number of novel variants (Fig 1C and 1D, S1 Table in S2 Material), both figures reflecting an isolated population previously unexplored by WGS data.

About 84% of SNVs previously reported in the WGS 12 NM project [16] were also detected here (S1 Fig in S1 Material). The unaccounted 16% SNVs could be attributed in part to technical differences in sequencing and variant calling, since the 12 NM were sequenced and processed with Complete Genomics proprietary technology and algorithms [29].

### Variants of interest in NM

Excluding singletons and private homozygous variants, we identified 35,105 novel SNVs and 9,013 novel indels (S6 Table in S2 Material) in the JPVS. These novel variants were not the result of low sequence coverage in other populations since about 94.1% of them were located at sites with more than 10X mean coverage in gnomAD 2.1 (S2 Note in S1 Material). According to LDlink 3.8 [30], 99.6% of novel variants are not currently surveyed by commonly used SNP-arrays, and 99.5% are not in linkage disequilibrium with known variants in the Mexican population. This novel variation dataset expands our knowledge of regional genomics, and should be included in future SNP-array designs aimed to massively explore population specific variation.

We found 12,871 potentially health related SNVs on average per individual (S2 Fig in S1 Material and S1 Table in S2 Material). Since common variants with allele frequency greater than 5% (AF > 5%) could be prioritized for large-scale genomic medicine studies, we subsetted the catalog by allele frequency and biomedical categories of interest. For example, we found 497 common SNVs mapped to some drug response gene according to PharmGKB [31] (Table 1). Another 20,408 common variants were associated with traits in the GWAS catalog [32], and 13,898 were related to clinical phenotypes in ClinVar [33]. On average each individual carried 8.4 homozygous variants with pathogenic or likely pathogenic clinical significance (ClinVar), without conflicting interpretations of pathogenicity, an assessment that should be reconsidered either taking into account the native Mexican origin of the individuals or the fact that we are reporting them in healthy individuals of old age.

**Table 1:** NM whole genome variation summary.

	SNV			INDEL		
	Rare (AF < = 1%)	Low (AF 1–5%)	Common (AF > 5%)	Rare (AF < = 1%)	Low (AF 1–5%)	Common (AF > 5%)
**ClinVar**	3.566	2.812	12.936	262	269	962
**Coding**	25.784	9.118	28.945	843	342	592
**dbSNP known**	1,918,765	1,128,329	4,930,622	225.923	211.317	592.412
**dbSNP novel**	0	34.791	314	0	7.148	1.865
**GeneHancer**	155.175	69.571	279.838	18.254	13.915	36.739
**GWAS catalog**	1.623	2.789	20.037	28	58	371
**PharmGKB**	28	66	497	0	0	0

AF = allele frequency.

We detected 1,865 novel indels and 314 novel SNVs with AF > 5%. Since the exome has been much more explored than WGS, most of these previously unreported frequent variants affect noncoding regions of the genome (Fig 2, S7 Table in S2 Material).

**Fig 3:**
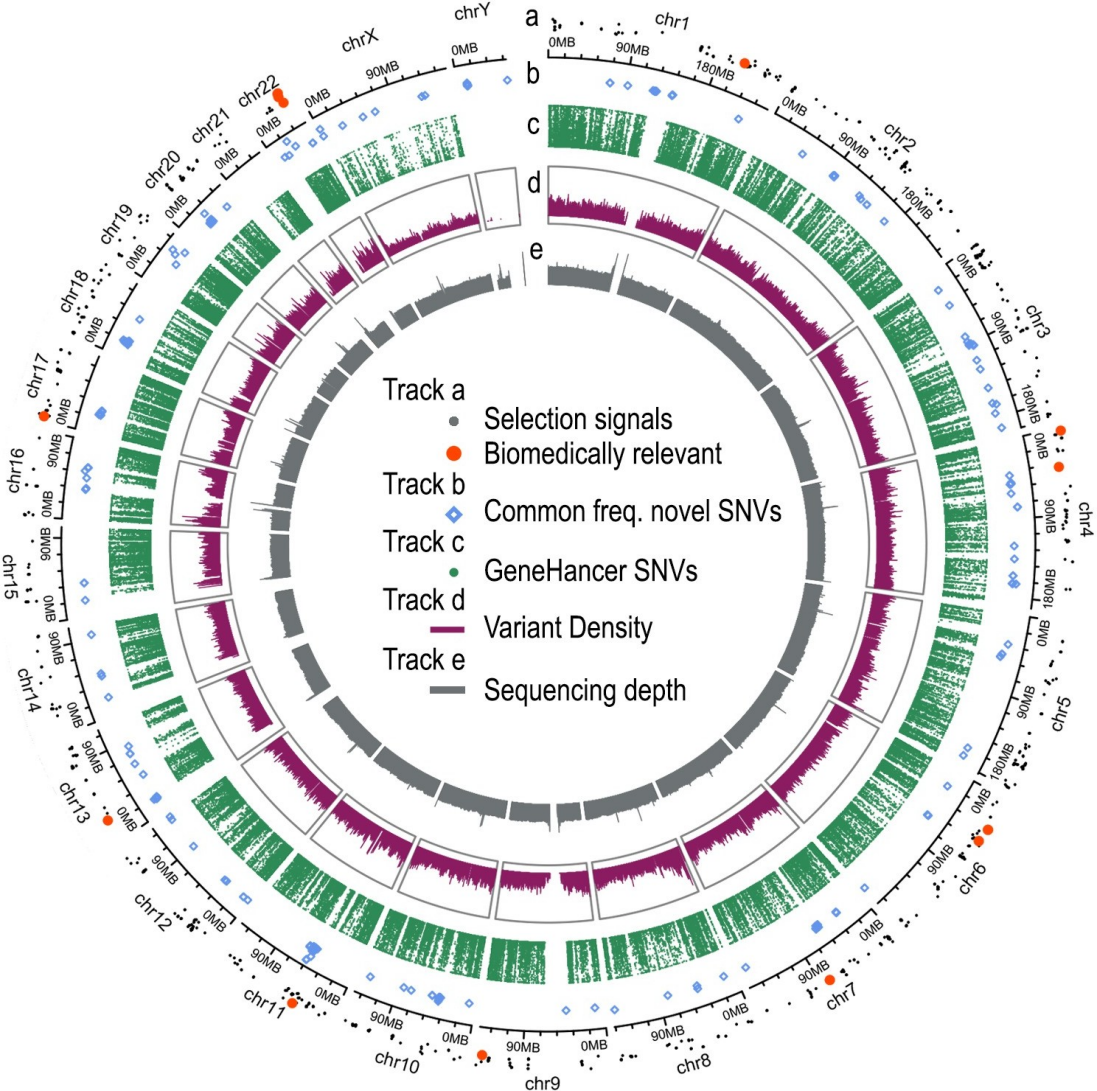
GRCh38 variation overview in NM. (a) SNVs under selection, health related selection signals (matching a GWAS catalog or ClinVar registry) are highlighted in orange. (b) Novel SNVs with allele frequency higher than 5%. (c) SNVs altering enhancer or promoter elements. Height of the dots in a, b and c depicts the allele frequency of the variants. (d) Population-wide variant density. (e) Average NGS genome coverage.

We also found 316,577 common variants (AF > 5%) in enhancer or promoter elements across the genome (Fig 3), which amount to approximately 5.7% of all the common variation (S3 Fig in S1 Material). Among the top genes with the most common variants in enhancer or promoter elements (S8 Table in S2 Material) we found: CMIP, a c-Maf inducing protein implicated in T-cell signaling and NCOR2, a transcriptional co-repressor implicated in chromatin structure modification. The subset of NM variation focused on the high quality set of regulatory elements provided by GeneHancer [34] represents potentially regulatory variants that could help to expand the study of population specific gene regulation.

Given the 2019 SARS-CoV-2 pandemic [35], we surveyed variation in the ACE2 receptor, a protein that enables viral entry into human cells, and found 86 variants, with only 2 synonymous and the rest of them noncoding, such that no structural changes in the receptor could explain any possible differential population susceptibility to SARS-CoV-2 infection in NM. However, it is worth noting that the large number of noncoding variants could influence ACE2 gene expression differences in these populations.

### Signals of positive natural selection

To identify signals of natural selection associated with SNVs in autosomes we used two complementary approaches: Population Branch Statistics (PBS) [36], a method for identifying variants with differential allele frequencies, and integrated Haplotype Score (iHS) [37], a method for detecting longer than expected haplotypes. With PBS we compared F_ST_ between our NM dataset, an ingroup composed by Peruvian samples with predominant NatAm ancestry, and an outgroup of Han Chinese samples. Alternatively, iHS allowed us to detect extended stretches of homozygosity surrounding loci of interest as a signal of selective sweeps in the NM dataset. We intersected results from PBS and iHS to pinpoint SNVs most likely to be under selection, since PBS can detect signals of selection [36], while iHS allows us to detect selective sweeps for very recent selection events [37, 38]. The intersection between both methods reduces false positive signals by taking into account the demographic history factor underlying the iHS analysis, a common confusion factor in FST based approaches [9, 37].

We identified 1,810 SNVs with selection signals distributed across the autosomes (Fig 3), with 0.8% located at coding regions (Fig 2, S9 Table in S2 Material). In conjunction with the explicit annotation of clinically relevant data, these signals of positive selection allowed us to focus the description of variants of interest from a population perspective.

Nonsynonymous selection signals were found in the following genes: MYO7B, a myosin required for the normal brush border action of intestinal and kidney microvilli [39]; BHLHE41, a transcriptional repressor implicated in sleep pattern control and circadian clock regulation [40]; CHRNE, a subunit of an acetylcholine receptor located at neuromuscular junctions [41]; MCHR1, a G protein-coupled receptor for melanin-concentrating hormone possibly involved in food consumption [42]; and PNPLA1, a phospholipase enzyme responsible for the biosynthesis of skin barrier lipids [43].

Regarding health related variants, we found signals of natural selection associated to the following traits previously identified in GWAS analysis [32] (S10 Table in S2 Material): increase of platelet count, increased lymphocyte counts, increased eosinophil percentage of white cells, increased cholesterol levels, increased blood pressure, increased body mass index, alcohol consumption, psychological depressive symptoms and cognitive performance.

We located 108 selection signals in enhancer or promoter sequences (S11 Table in S2 Material), with several enhancer-gene pairs carrying more than one SNVs under selection in these regulatory regions (S12 Table in S2 Material). Remarkably, the gene with more enhancer selection signals was PPARG, a nuclear receptor/transcription factor that controls lipid metabolism and adipogenesis [44]; PPARG has 10 selection signal SNVs across 3 enhancer elements, with a pattern of common allele frequencies, suggesting the existence of a haplotype that is absent from East Asians and shorter in other populations (S4 Fig in S1 Material and S13 Table in S2 Material). Since PPARG has previously been implicated in numerous diseases including obesity, diabetes, atherosclerosis [45], and insulin resistance in Mexican children [46], these variants merit a thorough follow-up analysis, including validation in a bigger, more diverse cohort, and experimental surveying of the variation effect on the regulated gene.

Other genes with multiple signals of natural selection in their regulatory regions are: ITPR2, an ion-gated channel related to glutamate-mediated neurotransmission with a role in apoptosis [47], and implicated in sweat production; IKBKE, a kinase involved in antiviral response signaling [48] and, also, a breast oncogene; SYN2, a synapsin implicated in neuronal vesicle trafficking whose variants are associated with autism, epilepsy, bipolar disorder and schizophrenia [49, 50]; and MKL1, initially identified as a Leukemia related gene, a transcription coactivator that controls smooth muscle cell differentiation in embryonic stem cells [51], myofibroblast formation, and adipocyte differentiation [52], previously identified as a marker for adaptation to climate conditions in North Eurasia [53], and closely related to PPARG function.

We performed gene set enrichment analyses focused on all genes with selection signals (S14 Table in S2 Material). An approach using Enrichr [54] reported significant overrepresentation of genes related to “Waist-to-hip ratio adjusted for BMI in non-smokers” (P-value = 1.9 x 10–5) according to GWAS catalog registries, while Key Pathway Advisor reported 24 enriched key pathways (S15 Table in S2 Material), most of them related to immune mechanisms.

We found 10 genes with signals of positive selection that were also reported in the analysis of 76 NM exomes [17], which are: CDH18, CHRNE, HNRNPH1, KLB, MCHR1, NCKAP5L, PYDC2, RABEP1, THEM6, UBXN2B; most of these genes are located at, or related to the function of cellular membranes.

### Genetic divergence between NM and global populations

To build an inter-population variant set (IPVS), we integrated the NM variant dataset with variants from 358 individuals of the 1000 genomes project [55] (S16 Table in S2 Material), including Han Chinese (CHB. From Beijing, China), Utah residents with Northern and Western European ancestry (CEU. Utah, USA), Yoruba (YRI. Ibadan, Nigeria), Peruvians with high NatAm ancestry (PEL. Lima, Perú), and admixed Mexicans (MXL. Los Angeles, USA). The IPVS includes 22.8 million SNVs with genotype information at every position (0% missing data), both in the NM and the international individuals.

For each SNV we calculated the proportion of heterozygous carrier individuals by population and then averaged those proportions for each population. This population heterozygosity ratio was lower in NM than other populations (Table 2). We analyzed haplotype block length [56] by calculating D confidence intervals in pairs of variants and then reconstructing LD blocks. The average haplotype block length in NM was 11.8 kb with a median of 3.4 kb, longer than CEU, CHB, YRI, MXL and PEL populations. The low heterozygosity and longer haplotype blocks in the NM compared to other world populations could be due to a low population size during the peopling of America, and also to the isolation of some communities.

**Table 2:** Heterozygosity ratio and haplotype block length per population.

Population	HET(avr)	HET(med)	Average Haplotype Length (kb)	Median Haplotype Length (kb)
**MXL**	0.3025	0.3175	6.2	2.108
**CEU**	0.2939	0.3125	6.13	2.079
**YRI**	0.2935	0.3165	1.703	669
**CHB**	0.2834	0.301	7.788	2.484
**PEL**	0.2735	0.2727	10.01	3.153
**NM**	0.2596	0.2763	11.89	3.488

Using the IPVS, we estimated intercontinental genetic structure by Principal Component Analysis (PCA), which showed a pattern similar to previous reports (S5 Fig in S1 Material) [57]. We observed that some PEL individuals of high NatAm ancestry clustered with NM. This prompted us to question what was the structure within America inferred with publicly available NatAm genomes.

**Fig 4:**
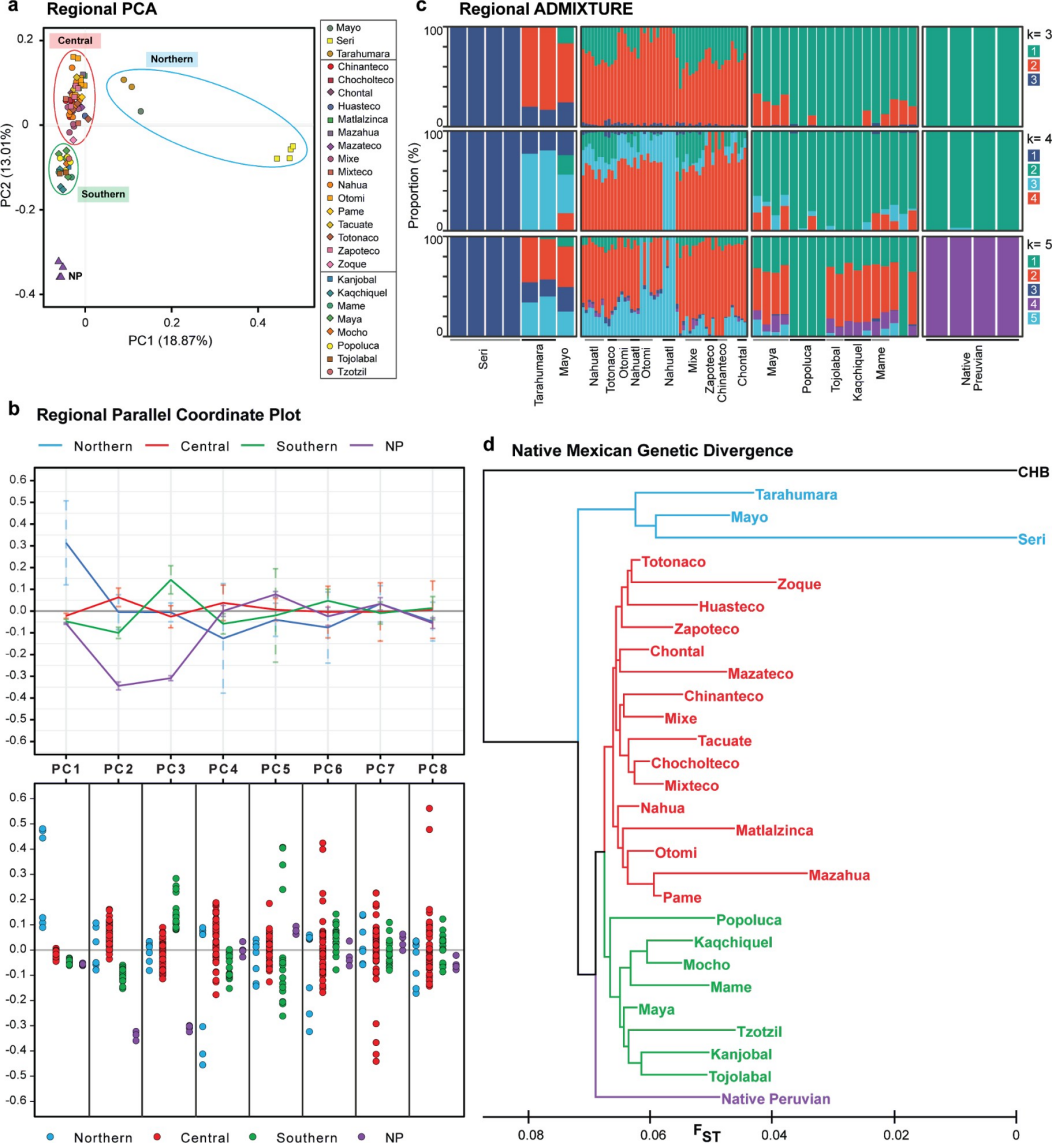
NM demography. (a) PCA of NM including 4 Native Peruvians (NP). (b) Summarized Parallel Coordinate Plot, showing only statistically significant PCs; (b) top panel, PC values per region, solid lines depict mean values, and dashed lines depict standard deviation; (b) bottom panel, dotted parallel coordinate plot, each dot depicts an individual. (c) ADMIXTURE analysis for different k, samples are ordered by geographic latitude and ethnic group. (d) Neighbor-joining tree based on F_ST_ between the 27 NM groups and NP in our study; colors indicate Region from Fig 4B.

Thus, we analyzed the local population structure of NM by PCA, including the four PEL individuals with NatAm ancestry higher than 95% (NP). The PC1 separated Seri from the rest of populations, while the Northern Tarahumara and Mayo clustered together; Central and Southern populations also clustered together (Fig 4A), while the NP individuals formed their own cluster. We proceeded to define the regionalization of individuals by an unsupervised k-means clustering analysis, as inferred from the 8 statistically significant PCs. Surveying a number of 5 groups (k = 5), Seri individuals formed their own cluster; NP also formed a particular cluster, with the remaining samples grouping in Northern, Central and Southern clusters (S3 Note in S1 Material). With this unsupervised regional clustering we tagged samples in a Northern-Central-Southern axis for the rest of the discussion (the Seri were included in the Northern group due to geographic location).

To simplify the regional patterns observed by PCA we calculated centroid values for each PC per geographic region. In PC2 and PC3 the NP separate on their own axes of variation (Fig 4B, top panel). In the rest of significant PC’s (which amount to 74.92% of the explained variance) the granular view of individual distribution shows that NM individuals have close values to NP (Fig 4B, bottom panel). These results show that NP do not exclusively differentiate from NM when taking most of the explained variance into account.

We performed ADMIXTURE analysis to explore the regional population structure in more detail. By analyzing the structure in k = 3, we observed a Northern-Central-Southern axis of genetic clustering, with the Seri once again being clearly distinctive; in k = 4, substructure was observed in the Central and Southern regions; in k = 5, NP form their own cluster. It is worth noting that the NP individuals closely share patterns of variation with the Southern individuals (Fig 4C), a concordant result with PC1 and PC2 from the PCA.

To identify population sub-clustering based on genomic variation similarity, we applied the k-means analysis running k values from 2 to 20. The most optimal clustering (highest silhouette index) occurs when k = 2 and the Seri form a group, with the second group embedding all the other individuals (S3 Note in S1 Material). Since this is clearly an effect of the Seri genomic context, we used k = 9 to identify subgroups. At this k the Central region shows the most subgroups, with the Southern region only subdividing in the Popoluca, and the rest of the groups (collectively known as the mayan cluster due to the linguistic family). Taking into account the PCA, ADMIXTURE, and k-means analysis, results suggest that the Native Peruvians are closer to the Mayan cluster than to the Central or Northern NM populations. A similar observation has been previously reported in a Peruvian genome project [58].

We further analyzed population substructure by measuring genetic divergence by F_ST_ between populations, and building a tree using a Neighbor-joining algorithm (Fig 4D). Results show that populations clustered according to their geographical origin, representing the Northern-Central-Southern axis, as previously reported for SNP-array data from 9 NM groups [3]. Phylogenetic relationships inferred by TreeMix also showed a pattern of geographic regionalization (S4 Note in S1 Material). It is worth mentioning that the close genomic similarity between the NP and the Southern groups was not observed in the Treemix representation; also, no gene flow was detected between said groups and NP. Thus, with the current evidence the similarities between NP and Southern NM observed by PCA and ADMIXTURE, cannot be attributed to a population split from the Central and Northern NM. In fact, we inferred a population split occurring between NP and NM, followed by a split of Southern, Central and Northern groups.

In both of our tree representations, the Seri show high values for genetic divergence. We confirmed this distinctive genomic context by identifying particular SNVs with high frequency bias for each NM indigenous group with at least four members, defined as variants with high frequency (AF > 0.5) in the group and rare (AF < 0.05) at worldwide scale. Seri showed 2,496 particular SNVs, 8 of them fixed, followed by Popoluca with 435, and none fixed (S6 Fig in S1 Material). The occurrence of these high frequency SNVs could be indicative of rapid diversification, isolation, and small population sizes.

This report is the largest compilation of NM genomic variation to date, complementing other whole genome [16] and exome [17] reports focused on Mexican genetic variation. Between these three projects, the genomic variation of 31 of the 68 indigenous groups distributed across Mexico has been studied by NGS. Further deep sequencing projects are needed to catalog the genomic variation of the remaining indigenous groups to complete the genomic landscape of NM. The NM variation catalog allowed us to explore variants subjected to natural selection, health phenotypes and the population structure, showing a clear Northern-Central-Southern axis. Seri is the most genetically distinct group, reflecting a small, isolated population, in concordance with anthropological information (Marlett 2011). It must be noted that our analysis of population structure has some limitations; the first one being that some NM groups are more represented than others, both in terms of numbers and geographic location, and the second is the inherent limitations of PCA and ADMIXTURE for structure definition, since there are other options that would allow us to explore fine-scale population structure. Both of these limitations can be overcome in future analyses with an increase in sample numbers by gathering and homogenizing all the available NM whole genomes, generating more sequences from the yet genomically unexplored NM groups, and applying improved analysis methods to make more precise demographic inferences.

The dataset from our project is a source for future research on personalized medicine in Mexico, and it is available upon request for scientific purposes in an effort to promote data sharing without compromising the genomic identity of the NM groups that participated in this study, a requirement for ethically fast tracking human genomics. With the current lack of representative NM whole genomes in the collective genomic knowledge, more projects like this one will be required to fill those gaps.

## Methods

### Ethics statement and sample collection

The study was performed in conformity with the Declaration of Helsinki, and approved by the Research, Ethics, and Biosafety Human Committees of the Instituto Nacional de Medicina Genómica (INMEGEN) in Mexico City (protocol number 31/2011/I). It was also performed with the support of the National Commission for the Development of Indigenous Communities (CDI, from the Spanish “Comisión Nacional para el Desarrollo de Pueblos Indígenas’’) and in agreement of the Indigenous leaders from each community. Each of the participants was guided through informed consent and signed the consent form. When it was necessary, informed consent was translated into the native language of the participant, and some individuals signed with their fingerprint. The 76 individuals belong to 27 indigenous groups across Mexico; these individuals are part of the Metabolic Analysis in an Indigenous Sample (MAIS) cohort collected between 2012 and 2018, where inclusion criteria and sample collection were first described [59].

### DNA sequencing and quality control

Whole genome sequencing was carried out with a paired-end, 150 bp long library configuration at the Beijing Genomics Institute in an Illumina HiSeq X Ten device. We performed read quality control after mapping to the reference genome (S6 Note in S1 Material. Quality Control in NGS data).

### Code availability

In general, the pipelines we provide in Methods use Nextflow [60] for controlling data flow, custom bash and R scripts (mainly using tidyverse, data table, and Circlize packages) [61], and bcftools [62] for data handling and analysis. Pipeline access is granted via github public repositories and https://www.protocols.io/.

### Read QC and trimming

The quality of raw paired fastq reads was evaluated with FastQC v0.11.4. We used Trimmomatic v0.39 [63] to remove Illumina adapters and keep reads with a minimum length of 70 bp and an average quality per base of 28 or higher; the following trimmomatic arguments were used: LEADING:28 SLIDINGWINDOW:5:28 MINLEN:70.

### Read alignment

GRCh38 genome reference was downloaded at 25/04/2017 from the GATK resource bundle (https://software.broadinstitute.org/gatk/download/bundle); the reference genome was indexed with SNAP v1.0beta.18 [21] using a seed size of 20 and enabling the parameter `-exact `. For each sample, trimmed reads were aligned to the reference genome using the SNAP aligner v1.0beta.18. Duplicated reads were marked with Sambamba v0.6.6 [64].

### Coverage comparison with gnomAD

We calculated the mean depth of coverage from the 76 BAM files, at single nucleotide resolution using bedtools [65]. This resulted in ~3 billion data points for coverage (data available upon request), which we summarized and compared against gnomAD 2.1 [22] coverage data lifted over to GRCh38 using Crossmap [66] (S17 Table in S2 Material).

### Short variant calling

Individual BAM files with marked duplicates were processed through the following GATK 3.8 tool set: 1) IndelRealigner for minimizing alignment artifacts caused by indels, 2) BaseRecalibrator for recalibrating Phred quality scores, 3) HaplotypeCaller to calculate genotype likelihoods. To integrate a raw variants dataset, all the individual gVCFs from HaplotypeCaller were genotyped together using the GenotypeGVCFs tool followed by recalibration of variant genotype likelihoods (VQSLOD) with the VQSR tool from GATK 3.8. Variants with a VQSLOD > = 99.0 were marked as PASS.

### Dataset definition

#### Joint Population Variant Set (JPVS)

Raw population data was filtered to keep only sites marked as PASS by VQSR, and with AN > = 150 (i.e. call rate > 98.5%). Multiallelic sites were split into biallelic records, using bcftools; this resulted in 9,737,152 variants, 8,638,130 SNVs and 1,099,022 indels.

#### Inter-population variant set (IPVS)

Biallelic SNVs from the JPVS dataset were intersected with SNVs from 358 international samples in the 1000 genomes project using bcftools (sample ids are provided in S16 Table in S2 Material). For sites with missing data in the 1000 genomes project we took information from the 1000 genomes CRAM files aligned to the human genome build GRCh38 (ftp://ftp.1000genomes.ebi.ac.uk/vol1/ftp/data_collections/1000_genomes_project/); for each CRAM sample file the regions with missing data were selected and re-formatted to BAM format using samtools v1.5 [67]. We then used the GATK best practices protocol to do a join variant calling with the NM samples. Additionally, for sites with no genetic variant detected in the NM samples, we set the genotype to 0/0 (homozygous reference) only in sites where every NM gVCF reported at least 5 sequencing reads supporting the reference allele; this resulted in 24,017,150 SNVs for downstream analyses with the IPVS.

### Relatedness determination

Using SNVs from the JPVS, we inferred kinship between pairs of NM samples using King v2.1.3 [68] with a threshold for kinship coefficient > 0.0442. Kinship coefficients are available in S18 Table in S2 Material.

### Comparison with other NM whole genome variation data

We compared the SNVs from the JPVS with the SNVs dataset from 12 NM genomes (12G) reported by Romero-Hidalgo et al. [16]. The 12G dataset was lifted over to GRCh38. Both datasets were subsampled to include only individuals common to both projects (Tarahumara, Nahua, Totonaca, Zapoteca and Maya groups), which amount to 10 samples from 12G and 26 samples from this project. We quantified shared variants between both projects with bcftools (S1 Fig in S1 Material).

### Variant annotation (dx.doi.org/10.17504/protocols.io.bkhvkt66 in protocols.io)

Variants were annotated with clinically relevant information using a pipeline that integrates custom databases to Variant Effect Predictor (the pipeline can be downloaded from https://github.com/Iaguilaror/nf-VEPextended). In brief, annotation of rsIDs was performed with bcftools using dbSNP b152 (fileDate = 20181015). The clinically relevant databases used were: 1) GeneHancer (January 2019, V2) [34] from UCSC Table Browser [69], 2) PharmGKB (var_drug_ann.tsv downloaded 06/14/2019) [31], 3) GWAS catalog (v1.0.2) [32], 4) ClinVar (clinvar_20190403) [33], 5) miRBase (release 22.1) [70], 6) gnomAD 2.1.1 for continental allele frequencies, and gnomAD 2.1 for worldwide sequencing coverage. If required, custom databases were converted to VCF format with coordinates from dbSNP b152 for GRCh38.

### Novel variants detection (dx.doi.org/10.17504/protocols.io.bkh7kt9n in protocols.io)

The pipeline used to build the novel dataset can be downloaded from https://github.com/Iaguilaror/nf-vcf-novel-dataset-builder. We used bcftools to remove singletons and private homozygous variants from the annotated JPVS, then we filtered variants to keep only sites with no rsID assigned by dbSNP b152 and no co-located variant in the “Existing_variation” field annotated by VEP. The same pipeline was also used to summarize gnomAD 2.1 sequencing coverage in novel variants.

To enable the identification of novel variants surveyed by SNP-arrays or in LD with known variants, coordinates for were lifted over to GRCh37/hg19 using Crossmap with the chainfile from hg38ToHg19.over.chain.gz from UCSC. To find variants in arrays, we used NIH’s LDlink 3.8 [30] SNPchip finder (https://ldlink.nci.nih.gov/?tab=snpchip). To find novel variants in LD we used LDlink 3.8 LDproxy (https://ldlink.nci.nih.gov/?tab=ldproxy) in API mode, using the MXL population from the 1000 genomes project as reference.

### Selection signals detection

To identify signals of positive selection associated with SNVs in autosomes we used two complementary approaches, following the protocol from Reynolds et al. [9]: the Population Branch Statistics (PBS) [36], and the integrated Haplotype Score (iHS) [37]. With PBS we performed a locus by locus pairwise comparison of F_ST_ with vcftools between our NM dataset, an ingroup composed by Peruvian samples with predominant NatAm ancestry (> 85%), and an outgroup of Han Chinese samples, both from the 1000 Genomes Project. Alternatively, iHS values were calculated for each population using hapbin. The top 1% significant results from both methods were intersected to pinpoint SNVs most likely to be under selection (S19 Table in S2 Material).

### Variant cataloging (dx.doi.org/10.17504/protocols.io.bkmzku76 in protocols.io)

The pipeline to catalog and quantify variants can be downloaded from: https://github.com/Iaguilaror/nf-VCF-cataloguer. In brief, we categorized the annotated JPSV into rare (AF < = 1%), low frequency (1 > AF < = 5%), common frequency (AF > 5%), and selection signal variants; each of these categories was further separated into novel or known variants, coding effect variants, variants in enhancer or promoter elements, and variants with registries in ClinVar, GWAS catalog or pharmGKB.

### Variant counting (dx.doi.org/10.17504/protocols.io.bkv6kw9e in protocols.io)

The pipeline used for counting variants, novel sites, and worldwide singletons per sample in the JPVS dataset can be downloaded from: https://github.com/Iaguilaror/nf-100GMX-variant-summarizer. Worldwide singletons were defined as heterozygous variants detected only in 1 NM sample, and not segregating in any other world population.

### Particular variants per indigenous groups

The pipeline for counting high frequency variants per group can be downloaded from: https://github.com/Iaguilaror/nf-100GMX-variant-summarizer. In brief, from the annotated JPVS we selected variants with allele frequencies < 0.05 in the world population with the highest known frequency, according to both the 1000 genomes project and the gnomAD whole genome data; then, for each NM group with at least 4 samples, we selected variants with AF > = 0.5 in the group and AF < 0.05 in the rest of the NM samples.

### Gene set enrichment analysis

Key Pathway Advisor (Clarivate Analytics) was carried out using the list of genes carrying SNVs with selection signals (S14 Table in S2 Material) and considering only processes with an estimated P-value equal to or less than 0.05 and Key Hubs with an estimated P-value equal to or less than 0.01. We also performed a gene set overrepresentation analysis using Enrichr with the same set of genes as input, and focusing on traits in the GWAS Catalog database 2019.

### Heterozygosity calculation

We calculated the proportion of heterozygous individuals per variant site in the IPVS. For this, we selected biallelic SNVs sites with MAF > 0.05. Then the proportion of heterozygous individuals per SNV was calculated with Plink [71] module ‘—hardy’, and the average proportion for each population was calculated.

### Haploblock reconstruction

From the IPVS, we selected SNVs with MAF > 0.025. VCF files were transformed to PLINK-bed files. Haplotype block reconstruction was performed on each population separately using the PLINK module—blocks, with a maximum block reconstruction window set at 500 kbp.

### Principal component analysis, k-means, and ADMIXTURE (dx.doi.org/10.17504/protocols.io.bkwbkxan in protocols.io)

The pipeline for running PCA, k-means, and ADMIXTURE from a single dataset can be downloaded: https://github.com/jbv2/VCF2PCP. In brief, from the IPVS, we kept only our NM samples and the 4 NP individuals from the 1000 genomes project (samples ids: HG01926, HG01938, HG01961, HG02272). We kept biallelic SNVs with a MAF > 0.05 with bcftools v1.9-220-gc65ba41, and removed variants in linkage disequilibrium (r2 > 0.85) with bcftools +prune plugin using parameters—window 2000bp—nsites-per-win 1. We transformed VCF files into Eigenstrat format. PCA was performed using Smartpca from Eigensoft v6.1.4 [72] requesting numoutevec: 20. We kept eigenvectors with P-value < 0.01, then recalculated the percentage of variability per eigenvector, being 100% the sum of the selected eigenvalues. In k-means analysis we calculated the Average Silhouette method to define optimal clustering. For ADMIXTURE v1.3 [20] analysis we used the—seed 43 parameter.

### Genetic divergence by F_ST_

From the IPVS we selected the NM, NP, and 4 CHB individuals from the 1000 genomes project (samples ids: NA18639, NA18640, NA18641, NA18642). We kept biallelic SNVs with a MAF > 0.05 with bcftools, and removed variants in linkage disequilibrium (r2 > 0.85) with bcftools +prune plugin using parameters—window 2000bp—nsites-per-win 1. Pairwise F_ST_ values were calculated by Smartpca from Eigensoft v6.1.4; calculated values were put in matrix format for MEGA X [73] to build a neighbour-joining tree.

## Supporting information

S1 Material (PDF)

S2 Material (XLSX)

S1 Data (DOCX)

S2 Data (ZIP)

S3 Data (ZIP)

S4 Data (ZIP)
