# Correction: Whole genome variation in 27 Mexican indigenous populations, demographic and biomedical insights

**DOI:** 10.1371/journal.pone.0269217

**Published:** 2022-05-25

**Authors:** Israel Aguilar-Ordoñez, Fernando Pérez-Villatoro, Humberto García-Ortiz, Francisco Barajas-Olmos, Judith Ballesteros-Villascán, Ram González-Buenfil, Cristobal Fresno, Alejandro Garcíarrubio, Juan Carlos Fernández-López, Hugo Tovar, Enrique Hernández-Lemus, Lorena Orozco, Xavier Soberón, Enrique Morett

In the Data Availability statement, the point of contact for genome sequence data access requests is incomplete. The point of contact should include Professor Lorena Orozco (lorozco@inmegen.gob.mx).
