# Supplementary material for: Whole genome variation in 27 Mexican indigenous populations, demographic and biomedical insights
Source: PLoS One. 2021 Apr 8;16(4):e0249773. doi: 10.1371/journal.pone.0249773 (PMC8031408; doi:10.1371/journal.pone.0249773)
Supplement: S1 Material — (PDF) [file pone.0249773.s001.pdf]

S1 Material

Supplementary Figs

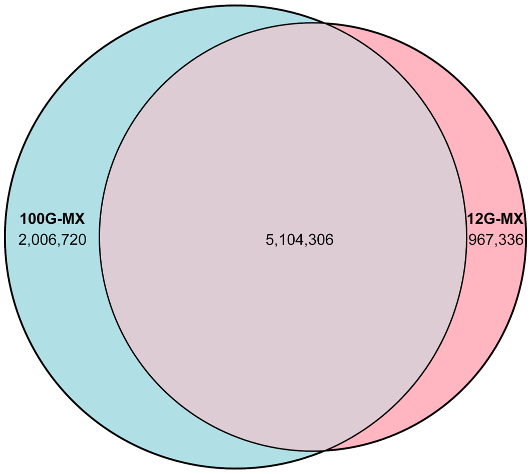

S1. Comparison our project (100G-MX) with the previous report of 12 Native Mexican WGS project. Number of SNVs is shown. We only compared individuals from ethnic groups shared by both projects: Tarahumara, Nahua, Totonaca, Zapoteca, Maya.

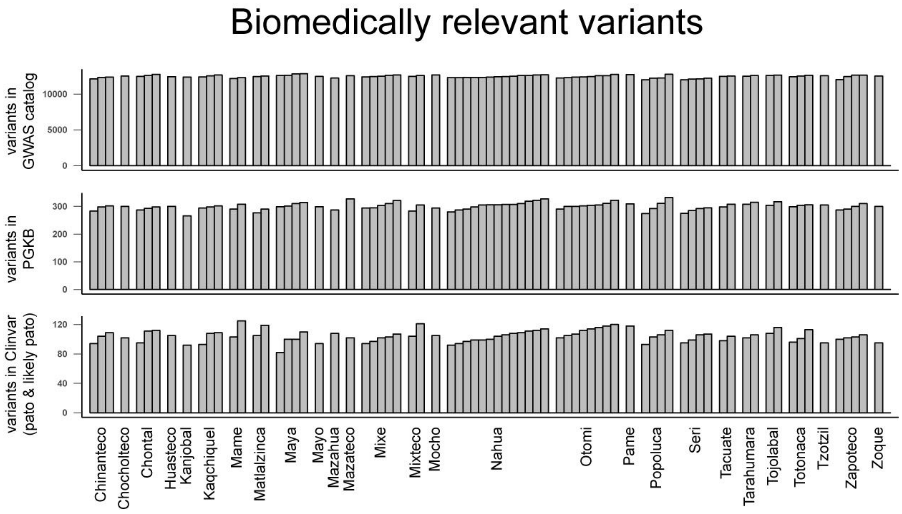

S2. Health related SNVs per sample. Annotation was performed using registries in the most updated releases (as of May 2019) of GWAS catalog, ClinVar and PharmGKB.

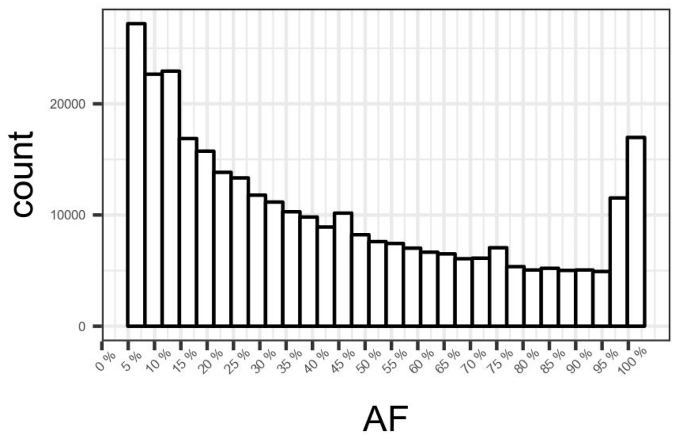

S3. Allele frequencies (as percentage) in common variants (AF > 5 %) found in enhancer or promoter elements.

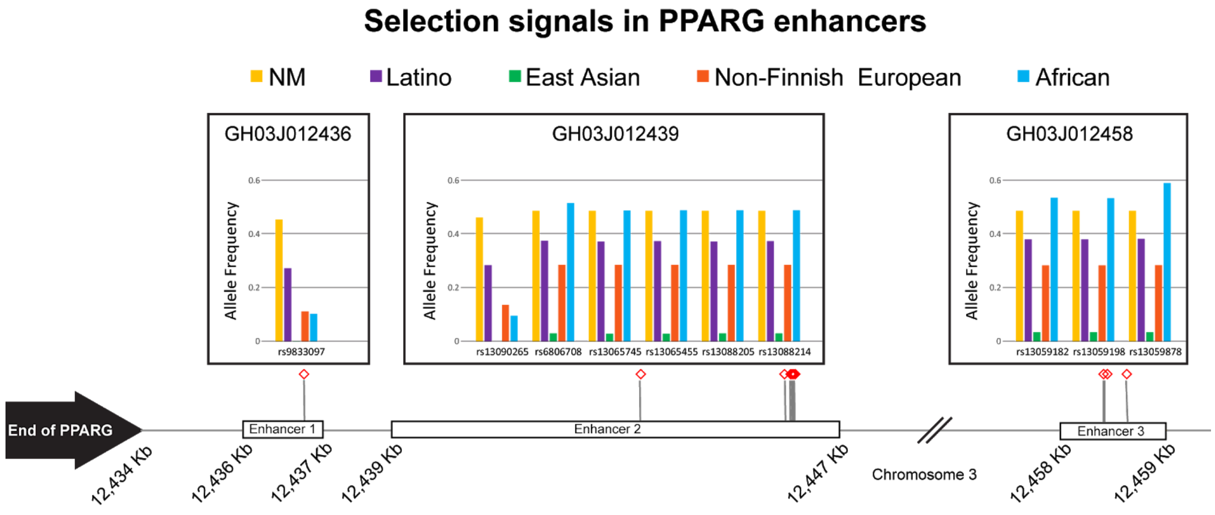

S4. Selection signal SNVs in PPARG. The pattern of Native Mexican common allele frequencies suggests the existence of an haplotype absent from East Asians and shorter in other populations.

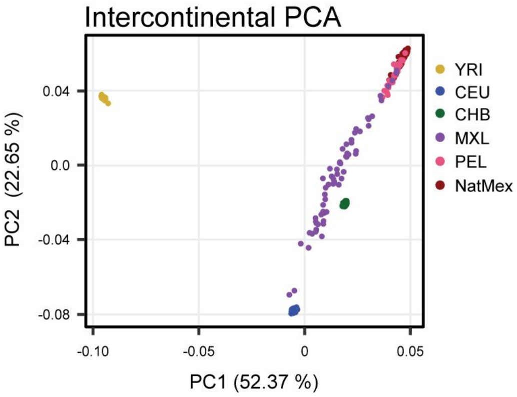

S5. Intercontinental PCA genetic divergence between Yoruba (YRI), Europeans (CEU), Chinese (CHB), Mexicans (MXL), Peruvians (PEL), and Native Mexicans (NatMex).

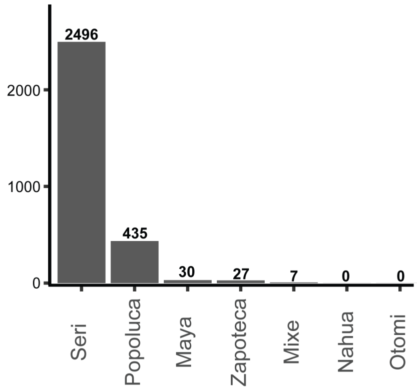

S6. High frequency biased SNVs per ethnic group. Only groups with at least four samples were included in the analysis. Particular variants were defined as those with an allele frequency of at least 50 % in the selected ethnic group, while also being in less than 5 % of the rest of the NM, and lower than 0.5 % in the highest reported population worldwide (as reported by VEP for 1000 genomes populations and gnomAD 2.1 whole genome data).

Supplementary Note 1. Ancestry in original samples.

We used 85% Native American ancestry as a threshold to include more samples in the dataset, only a few individuals showed less than 90% (Fig SN1). We explored the geographic distribution of Native American Ancestry in the dropped individuals and found an expected pattern of admixed genome, the Northern individuals being

more european, followed by the Central and Southern individuals. The non-uniformity of admixed ancestry in the country resulted in dropping 4 Northern individuals, but the remaining 7 still represent populations previously unexplored by WGS (Seri, Tarahumara, and Mayo).

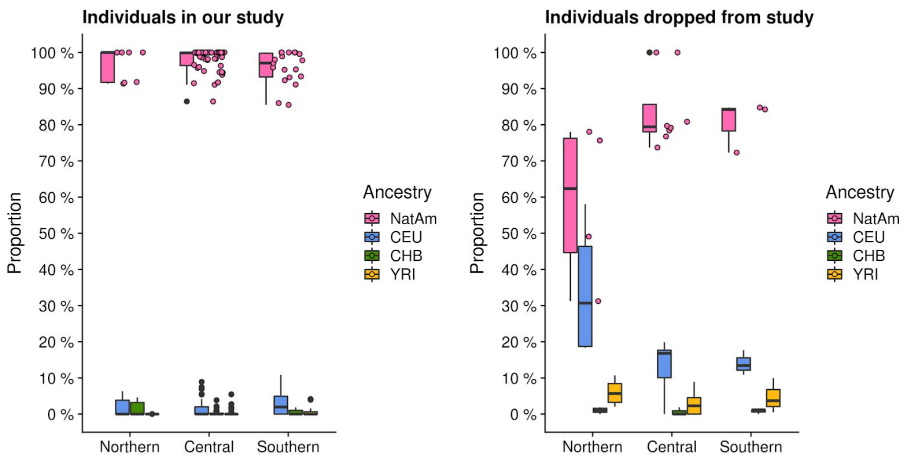

Fig SN1. Ancestry comparison between individuals included or dropped from the study. Individuals included in the study have a high mean proportion of Native American (NatAm) ancestry. In contrast, individuals dropped from the study have varying proportions of European (CEU), East Asian (CHB) and African (YRI) ancestry. The threshold to drop individuals from the study was 85 % Native American ancestry; the Central individuals with high Native ancestry were dropped due to relatedness with other included samples. Ancestry was calculated as described in the main text of this paper.

Supplementary Note 2. Coverage comparison with gnomAD.

We calculated the mean depth of coverage from the 76 BAM files, at single nucleotide resolution in the GRCh38 genome using bedtools. This resulted in ~3 billion data points for coverage (data available upon request); to summarize this data, we aggregated the mean depth in 100 kb windows across the whole genome.

Since gnomAD 2.1 [1] already provides mean depth of coverage at single nucleotide resolution for each base of the GRCh37 genome version, we lifted over this data to GRCh38 using Crossmap; then we calculated the mean depth of coverage in the same 100 kb windows of the GRCh38 genome as we did for our project's coverage.

Supplementary Table 17 compiles the mean depth of coverage comparison between our project and gnomAD 2.1 across every window of the GRCh38 genome. To quantify the genome fraction exclusively covered by our project, we added the window length of regions where our project had coverage > 0 but gnomAD coverage was 0 (Supplementary Table 4). In summary, we covered 2,936,850,045 bases of the GRCh38 genome (95.09 % of the reference file), with a mean depth of coverage of 24.08 X; while gnomAD 2.1 covers 2,836,240,773 bases (91.83 % of the reference file), with a mean depth of 29.9 X.

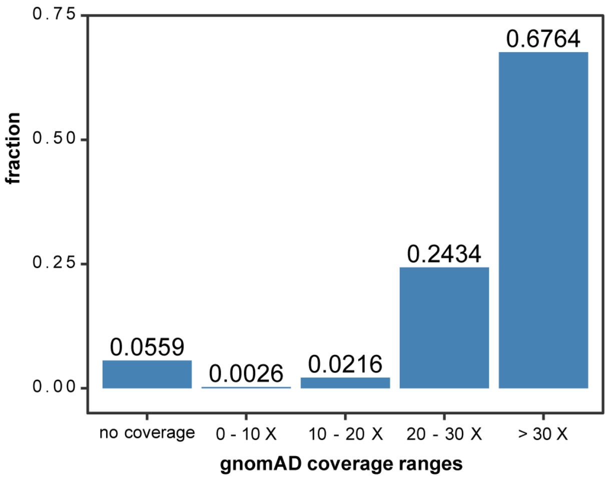

Fig SN2. Fractions of novel variants (SNVs and indels) from our study grouped by the corresponding coverage in gnomAD 2.1. For indels, we calculated the average coverage across every affected nucleotide in the reference.

Supplementary Note 3. Population structure of Native Mexicans

We explored the particular structure of NM and NP populations by PCA, using the IPVS with only NM and 4 PEL samples with Native American ancestry > 95% (as reported by the 1000 Genomes Project in: http://ftp.1000genomes.ebi.ac.uk/vol1/ftp/technical/working/20140818_ancestry_deconvolution/). We filtered the data to keep only biallelic SNPs with MAF > 0.05, and pruned variants by linkage disequilibrium. The remaining 647,478 SNPs were used as input for EIGENSOFT's smartpca. A parallel coordinate plot overview of the first 20 PCs shows the heterogeneity of individuals in our datasets (Fig SN 3.1). Only the first 8 PCs were statistically significant (p value < 0.01).

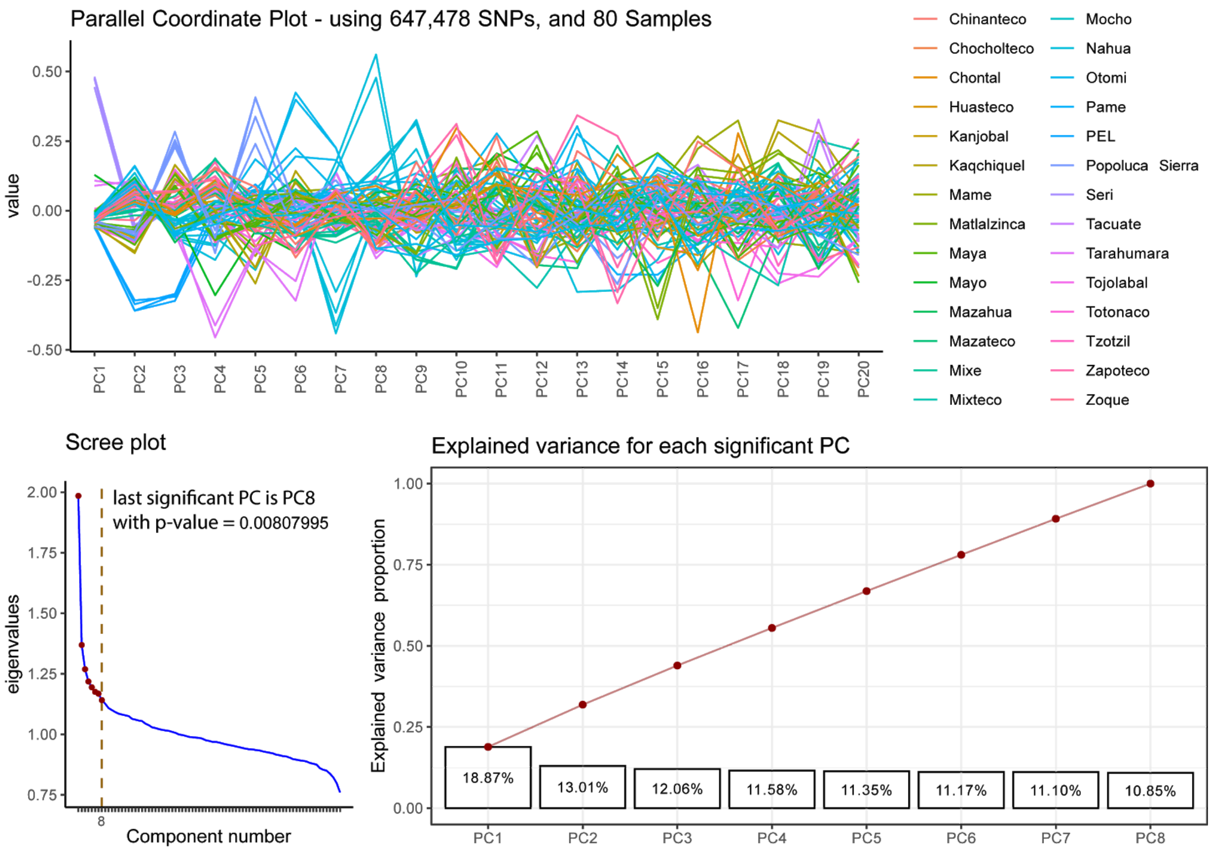

Fig SN3.1. Overview of Regional Principal Component Analysis. **Top panel**, Multi-individual parallel coordinate plot depicting every value for every PC. **Bottom left panel**, Scree plot indicating the last

significant PC (orange dashed line) with a p value < 0.01. **Bottom right panel**, explained variance for every significant PC; red line depicts cumulative variance.

We performed an unsupervised k-means clustering analysis. Using the 8 significant PCs, and a number of 5 groups (k = 5) we found that the Seri individuals are so distinctive that they form their own cluster; NP also form a particular cluster, with the remaining samples grouping in Northern, Central and Southern clusters (Fig SN 3.2). This unsupervised regional clustering was used to group samples in a Northern-Central-Southern axis for the discussion in the main text (the Seri, Mayo and Tarahumara were gathered in the Northern group due to geographic location).

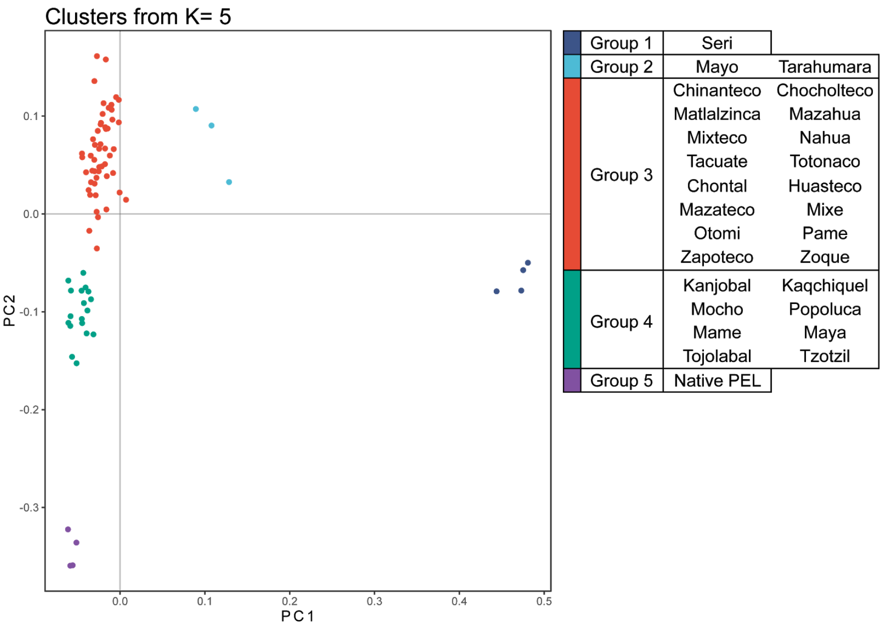

Fig SN 3.2. K-means analysis for k = 5. NM and NP can be clustered in 5 groups based on whole genome PCA analysis, The patterns of variation between individuals of the same group are similar, while being dissimilar between different groups.

We identified population subclustering based on genomic variation similarity by applying a k-means analysis, running k values from 2 to 20. We measured the quality of clustering by the Average Silhouette (Avg. Silh.) method, where a high

Avg. Silh. value indicates an optimal number of clusters. Unsurprisingly, the most optimal clustering occurs when k = 2 and the Seri form a group, with the second group embedding all the other individuals (Fig SN 3.3).

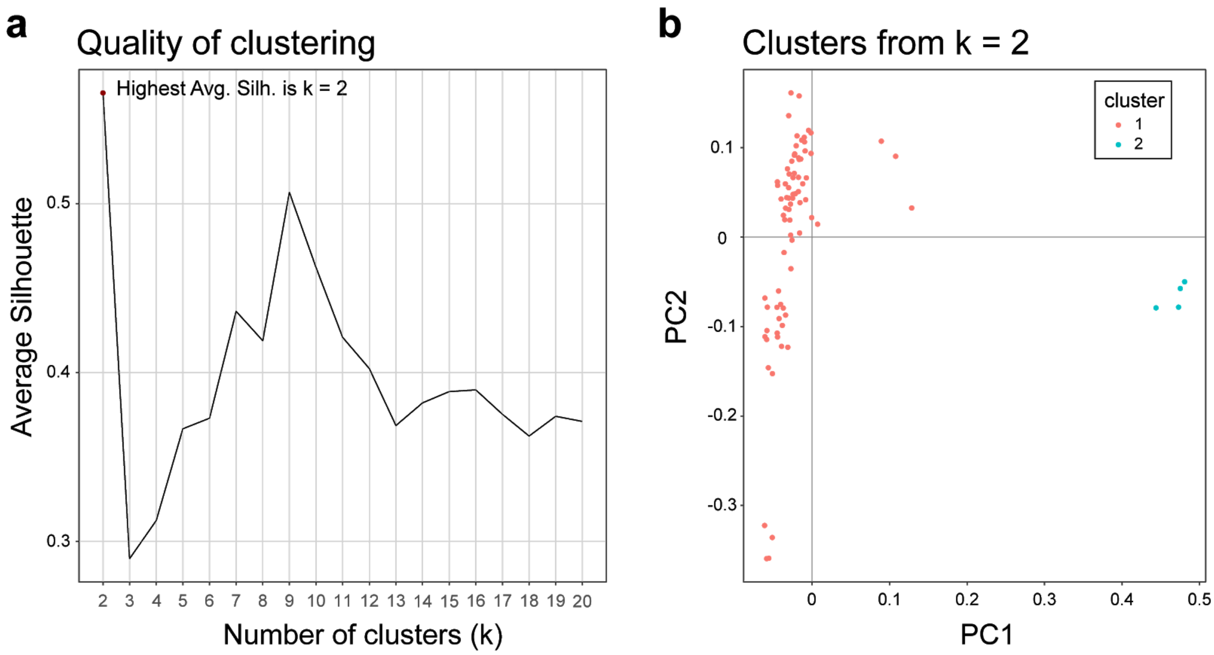

Fig SN 3.3. Population subclustering based on k-means. **a**, optimal clustering at different k values, measured by the Average silhouette method. **b**, the most optimal clustering in the NM dataset subdivides the Seri (group 2) from the rest of the NM and NP¨individuals (group 1), due to the distinctive Seri genomic context.

Since this is clearly an effect of the Seri genomic context, we chose the next highest Avg. Silh. at k = 9 to identify optimal subgroups (Fig SN 3.4).

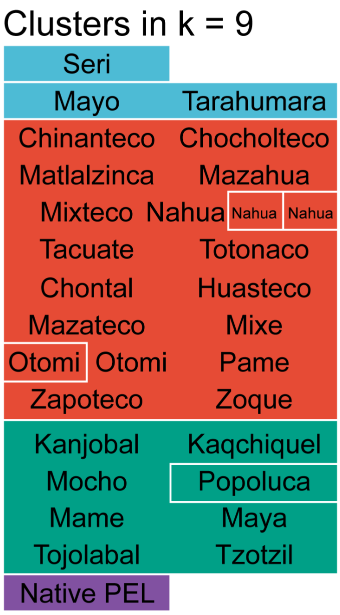

Fig SN 3.4. Optimal clusters in Native Mexican populations. Geographic regions are identified by colors: blue (Northern), red (Central), and green (Southern). The 9 clusters defined by the Average Silhouette method are marked as boxes, embedding ethnic groups belonging to each cluster. The Nahua subgrouping reflects the different location of the individuals; the same occurs in the Otomi subgroups.

Supplementary Note 4. Phylogenetic relationships in Native Mexicans

We built maximum likelihood trees inferred by TreeMix to represent phylogeny in Native Mexican groups in our study, including the Native Peruvians, and an East Asian individual as outlier. We inferred the tree with 0, 1 and 2 migration events. The tree topologies show the Native Peruvian branch separated from the main Native Mexican branch, and regional branchings similar with the structure detected by F_{ST} in the main discussion. Tree topologies coincide with previous reports on Native Mexican population substructure [2,3]. We also measured f3 statistics, with a Z score < -2 as an indication of gene flow between populations. We only detected signals between the East Asian outgroup and Central groups, and Maya Mayo and

Tojolabal. No gene flow signals were detected between Native Peruvians and Native Mexicans.

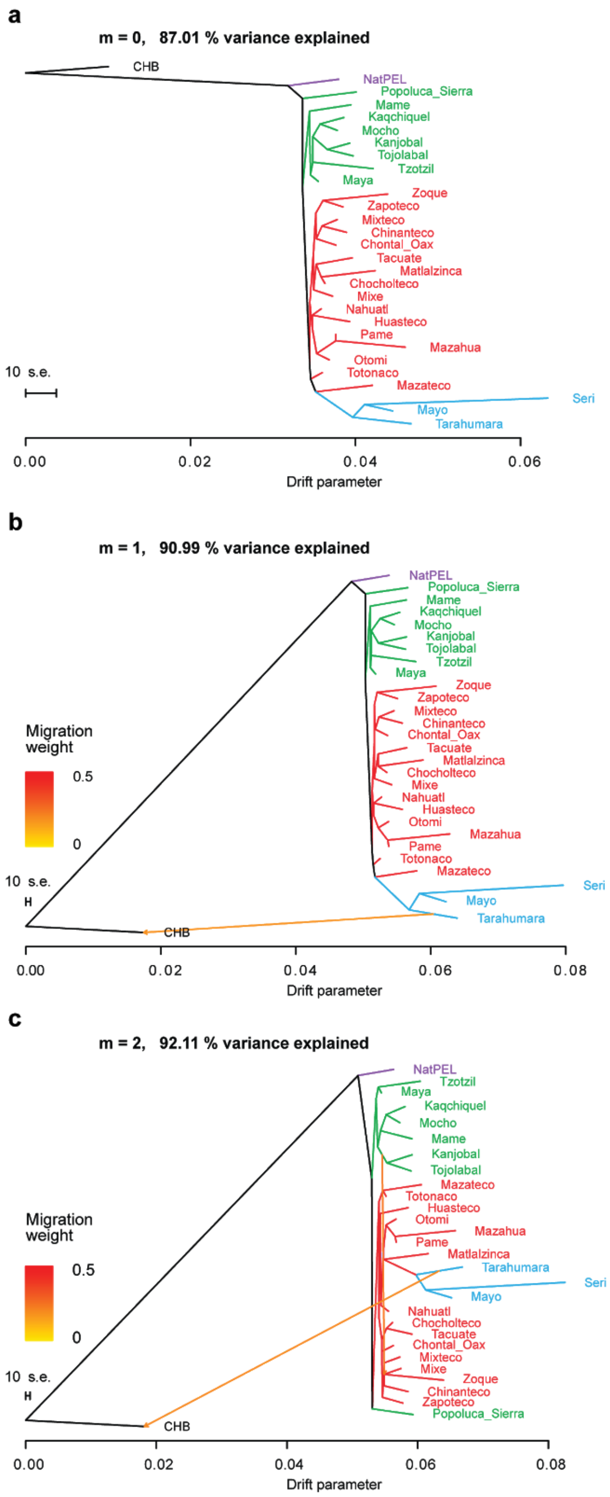

Fig SN 4.1. Phylogenetic relationships in Native Mexicans. For each panel we show the tree topology inferred by Treemix, with different migration events (m=X), branch length is measured by the x axis as the amount of genetic drift; scales show 10 times the average of standard error in the covariance matrix used to build the trees; arrows in the plot indicate migration events with gene flow direction. **a,** topology inferred with no migration event. **b**, topology inferred with 1 migration event. **c,** topology inferred with 2 migration events. The detected migration event between Tarahumara and CHB in B and C, coincides with previous reports [3].

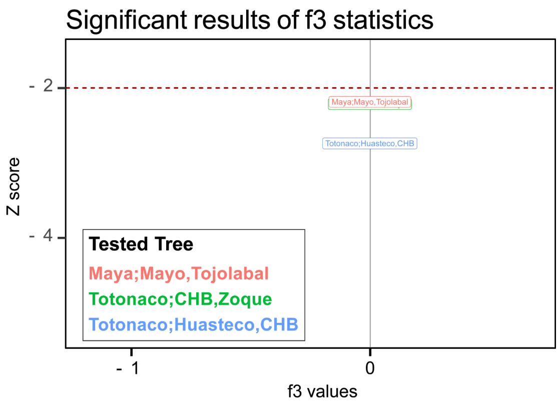

Fig SN 4.2. Gene flow in the studied groups, measured by f3 statistics. Red line indicates cutoff to define significant Z score values.

Supplementary note 5. Heterozygosity analysis

We calculated the intra individual heterozygosity for the Native Mexicans (NatMX) and the reference world populations CEU, YRI, CHB, MXL and PEL using the Inter-population variant set (IPVS). We selected only SNVs variants, sites without missiness and removed singletons. Then for each sample we calculated the ratio of homozygous variants and heterozygous variants (Figure SN 5.1). We showed using this approach that NatMX have the lowest heterozygosity ratio compared to other populations. MXL and PEL individuals with high Native American ancestry display the same level of heterozygosity as the NatMX (Figure SN 5.2). The Seris display the lowest amount of heterozygosity.

In order to check the effect of the admixture inside the NatMXs, MXLs and PELs against the heterozygosity ratio; we measured by sample without removing singletons, the heterozygosity ratio and Native American ancestry (Figure SN 5.2). Native American ancestry proportions in PELs and MXLs was obtained from the 1000 genomes project repository (https://www.internationalgenome.org/), Native American ancestry of the NatMX was inferred using admixture as described in the methods section. The heterozygosity ratio correlates negatively (pearson r= -0.588, p.val = $1.551e^{-08}$) with the level of Native American ancestry for the PELs and MXLs, the same in NatMX (pearson r= -0.913, p.val < $2.2e^{-16}$).

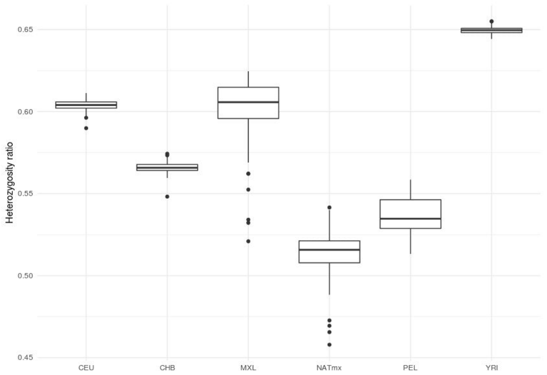

Fig SN 5.1: Heterozygosity ratio (number of heterozygous variants divided by the number of homozygous variants) in each sample from different populations.

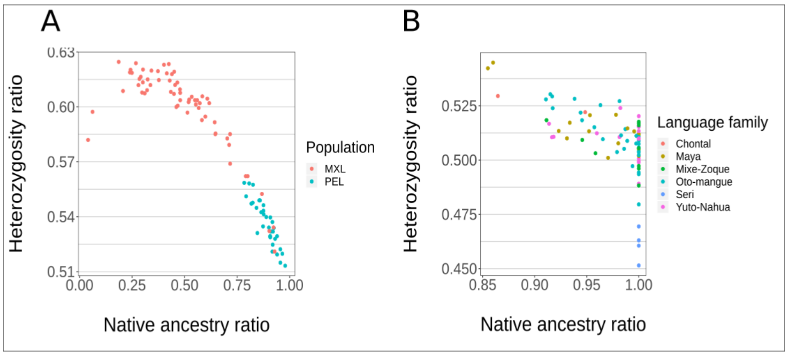

Fig SN 5.2: Heterozygosity ratio and native ancestry ratio. A) Ratios in MXLs and PELs (pearson r= -0.588, p.val = $1.551e^{-08}$), B) Ratio in NatMX (pearson r= -0.913, p.val < $2.2e^{-16}$).

Supplementary Note 6. Quality Control in NGS data

Here we show the NGS QC for the 95 samples originally sequenced for the project. We must note that some of those samples were not included in the main report. The full Qualimap report can be downloaded at:

https://drive.google.com/file/d/1BEMPtksCPpiC_oE3mtytOk0JNqQ9drSs/view?usp=sharing

Qualimap Analysis Results

Multi-sample BAM QC analysis
Generated by Qualimap v.2.2.1
2017/07/31 16:49:22

Global QC

**Number of samples**	**95**
**Total number of mapped reads**	**50,726,214,735**
**Mean samples coverage**	**22.32**
**Mean samples GC-content**	**42.7**
**Mean samples mapping quality**	**32.35**
**Mean samples insert size**	**322.46**

In the next pages we include general QC plots for parameters of interest. Plot titles describe the data shown, for each of the 95 original samples.

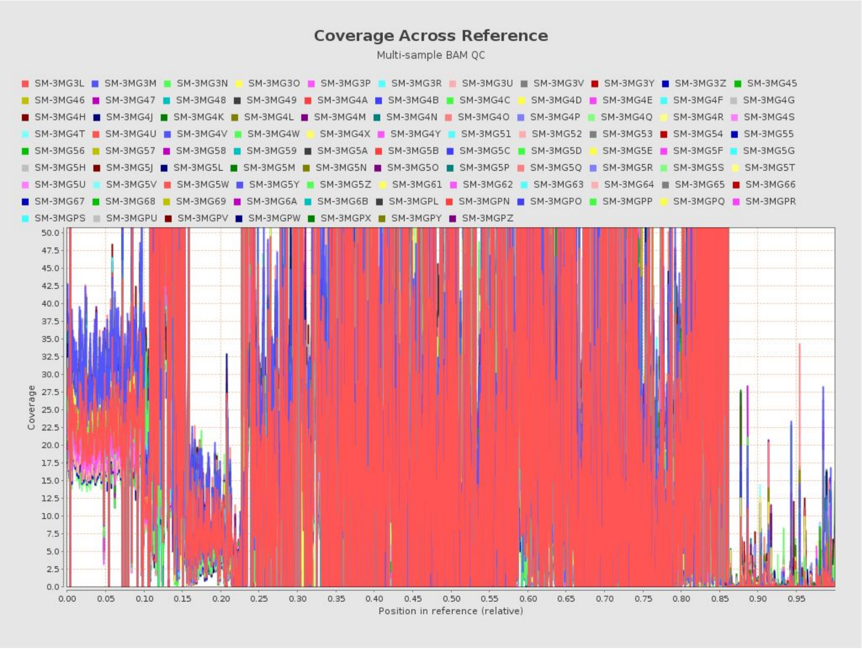
Coverage Across Reference
Multi-sample BAM QC
SM-3MG3L SM-3MG3M SM-3MG3N SM-3MG3O SM-3MG3P SM-3MG3R SM-3MG3U SM-3MG3V SM-3MG3Y SM-3MG3Z SM-3MG45
SM-3MG46 SM-3MG47 SM-3MG48 SM-3MG49 SM-3MG4A SM-3MG4B SM-3MG4C SM-3MG4D SM-3MG4E SM-3MG4F SM-3MG4G
SM-3MG4H SM-3MG4J SM-3MG4K SM-3MG4L SM-3MG4M SM-3MG4N SM-3MG4O SM-3MG4P SM-3MG4Q SM-3MG4R SM-3MG4S
SM-3MG4T SM-3MG4U SM-3MG4V SM-3MG4W SM-3MG4X SM-3MG4Y SM-3MG51 SM-3MG52 SM-3MG53 SM-3MG54 SM-3MG55
SM-3MG56 SM-3MG57 SM-3MG58 SM-3MG59 SM-3MG5A SM-3MG5B SM-3MG5C SM-3MG5D SM-3MG5E SM-3MG5F SM-3MG5G
SM-3MG5H SM-3MG5J SM-3MG5L SM-3MG5M SM-3MG5N SM-3MG5O SM-3MG5P SM-3MG5Q SM-3MG5R SM-3MG5S SM-3MG5T
SM-3MG5U SM-3MG5V SM-3MG5W SM-3MG5Y SM-3MG5Z SM-3MG61 SM-3MG62 SM-3MG63 SM-3MG64 SM-3MG65 SM-3MG66
SM-3MG67 SM-3MG68 SM-3MG69 SM-3MG6A SM-3MG6B SM-3MGPL SM-3MGPN SM-3MGPO SM-3MGPP SM-3MGPQ SM-3MGPR
SM-3MGPS SM-3MGPU SM-3MGPV SM-3MGPW SM-3MGPX SM-3MGPY SM-3MGPZ
Coverage
Position in reference (relative)

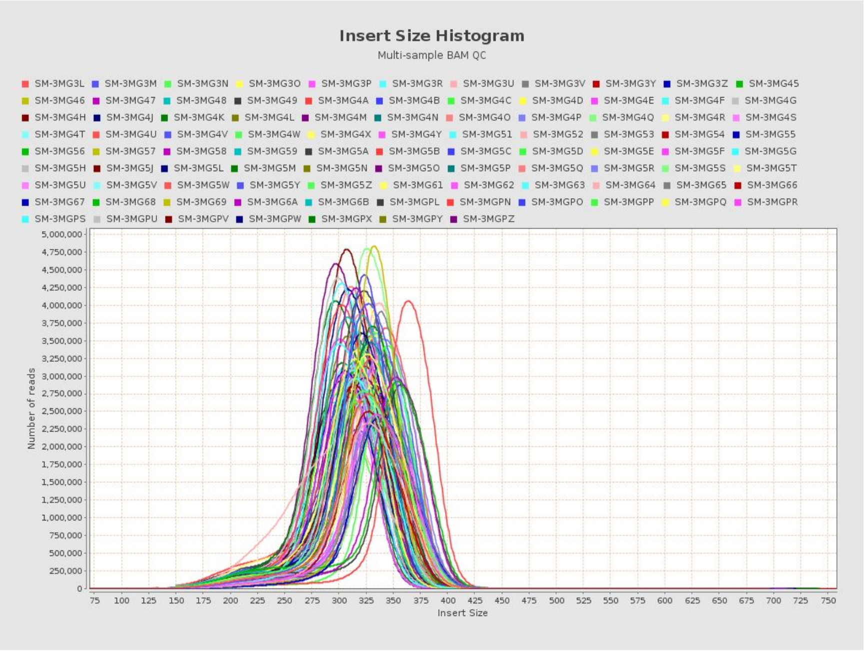
Insert Size Histogram
Multi-sample BAM QC
SM-3MG3L SM-3MG3M SM-3MG3N SM-3MG3O SM-3MG3P SM-3MG3R SM-3MG3U SM-3MG3V SM-3MG3Y SM-3MG3Z SM-3MG45
SM-3MG46 SM-3MG47 SM-3MG48 SM-3MG49 SM-3MG4A SM-3MG4B SM-3MG4C SM-3MG4D SM-3MG4E SM-3MG4F SM-3MG4G
SM-3MG4H SM-3MG4J SM-3MG4K SM-3MG4L SM-3MG4M SM-3MG4N SM-3MG4O SM-3MG4P SM-3MG4Q SM-3MG4R SM-3MG4S
SM-3MG4T SM-3MG4U SM-3MG4V SM-3MG4W SM-3MG4X SM-3MG4Y SM-3MG51 SM-3MG52 SM-3MG53 SM-3MG54 SM-3MG55
SM-3MG56 SM-3MG57 SM-3MG58 SM-3MG59 SM-3MG5A SM-3MG5B SM-3MG5C SM-3MG5D SM-3MG5E SM-3MG5F SM-3MG5G
SM-3MG5H SM-3MG5J SM-3MG5L SM-3MG5M SM-3MG5N SM-3MG5O SM-3MG5P SM-3MG5Q SM-3MG5R SM-3MG5S SM-3MG5T
SM-3MG5U SM-3MG5V SM-3MG5W SM-3MG5Y SM-3MG5Z SM-3MG61 SM-3MG62 SM-3MG63 SM-3MG64 SM-3MG65 SM-3MG66
SM-3MG67 SM-3MG68 SM-3MG69 SM-3MG6A SM-3MG6B SM-3MGPL SM-3MGPN SM-3MGPO SM-3MGPP SM-3MGPQ SM-3MGPR
SM-3MGPS SM-3MGPU SM-3MGPV SM-3MGPW SM-3MGPX SM-3MGPY SM-3MGPZ
Number of reads
Insert Size

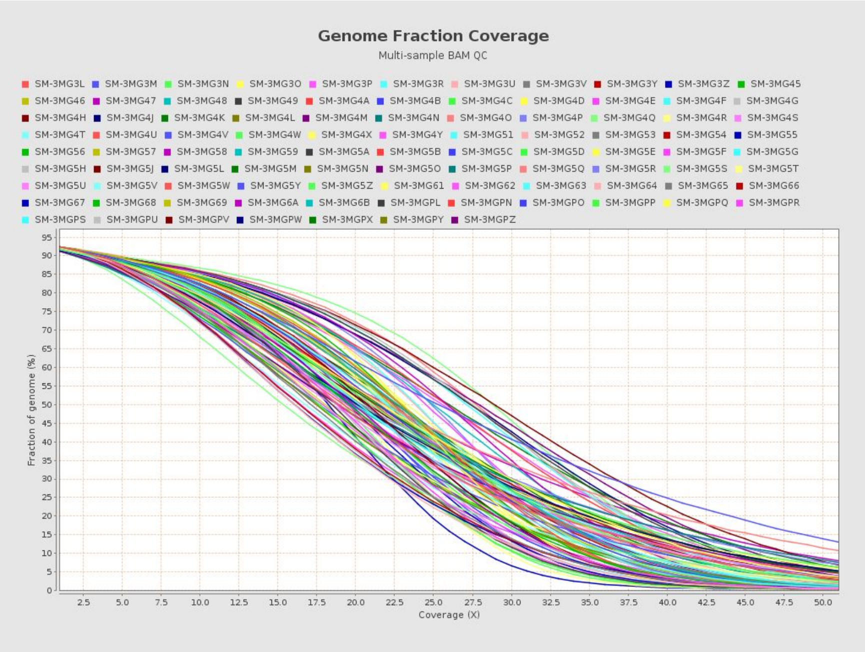
Genome Fraction Coverage
Multi-sample BAM QC
SM-3MG3L SM-3MG3M SM-3MG3N SM-3MG3O SM-3MG3P SM-3MG3R SM-3MG3U SM-3MG3V SM-3MG3Y SM-3MG3Z SM-3MG45
SM-3MG46 SM-3MG47 SM-3MG48 SM-3MG49 SM-3MG4A SM-3MG4B SM-3MG4C SM-3MG4D SM-3MG4E SM-3MG4F SM-3MG4G
SM-3MG4H SM-3MG4J SM-3MG4K SM-3MG4L SM-3MG4M SM-3MG4N SM-3MG4O SM-3MG4P SM-3MG4Q SM-3MG4R SM-3MG4S
SM-3MG4T SM-3MG4U SM-3MG4V SM-3MG4W SM-3MG4X SM-3MG4Y SM-3MG51 SM-3MG52 SM-3MG53 SM-3MG54 SM-3MG55
SM-3MG56 SM-3MG57 SM-3MG58 SM-3MG59 SM-3MG5A SM-3MG5B SM-3MG5C SM-3MG5D SM-3MG5E SM-3MG5F SM-3MG5G
SM-3MG5H SM-3MG5J SM-3MG5L SM-3MG5M SM-3MG5N SM-3MG5O SM-3MG5P SM-3MG5Q SM-3MG5R SM-3MG5S SM-3MG5T
SM-3MG5U SM-3MG5V SM-3MG5W SM-3MG5Y SM-3MG5Z SM-3MG61 SM-3MG62 SM-3MG63 SM-3MG64 SM-3MG65 SM-3MG66
SM-3MG67 SM-3MG68 SM-3MG69 SM-3MG6A SM-3MG6B SM-3MGPL SM-3MGPN SM-3MGPO SM-3MGPP SM-3MGPQ SM-3MGPR
SM-3MGPS SM-3MGPU SM-3MGPV SM-3MGPW SM-3MGPX SM-3MGPY SM-3MGPZ
Fraction of genome (%)
Coverage (X)

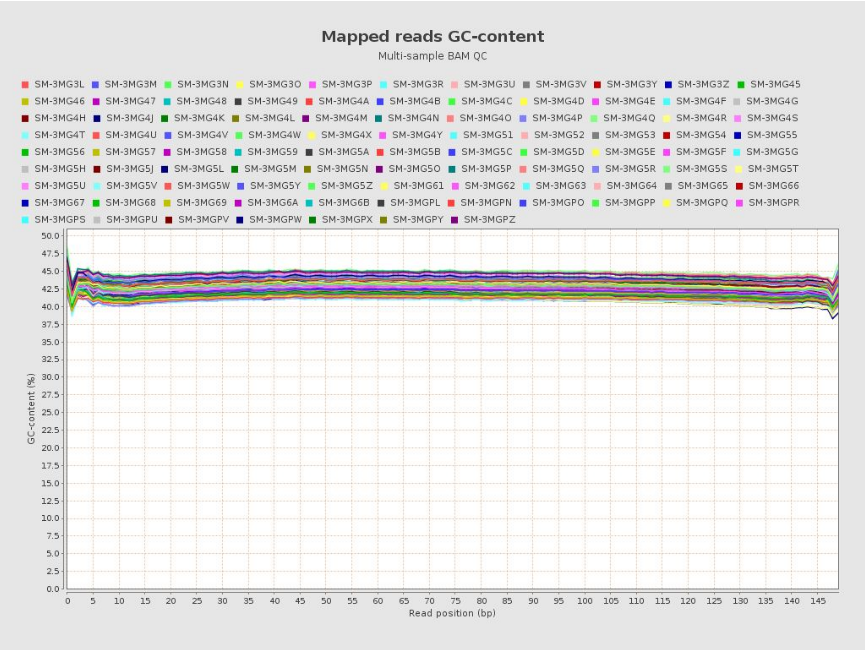
Mapped reads GC-content
Multi-sample BAM QC
SM-3MG3L SM-3MG3M SM-3MG3N SM-3MG3O SM-3MG3P SM-3MG3R SM-3MG3U SM-3MG3V SM-3MG3Y SM-3MG3Z SM-3MG45
SM-3MG46 SM-3MG47 SM-3MG48 SM-3MG49 SM-3MG4A SM-3MG4B SM-3MG4C SM-3MG4D SM-3MG4E SM-3MG4F SM-3MG4G
SM-3MG4H SM-3MG4J SM-3MG4K SM-3MG4L SM-3MG4M SM-3MG4N SM-3MG4O SM-3MG4P SM-3MG4Q SM-3MG4R SM-3MG4S
SM-3MG4T SM-3MG4U SM-3MG4V SM-3MG4W SM-3MG4X SM-3MG4Y SM-3MG51 SM-3MG52 SM-3MG53 SM-3MG54 SM-3MG55
SM-3MG56 SM-3MG57 SM-3MG58 SM-3MG59 SM-3MG5A SM-3MG5B SM-3MG5C SM-3MG5D SM-3MG5E SM-3MG5F SM-3MG5G
SM-3MG5H SM-3MG5J SM-3MG5L SM-3MG5M SM-3MG5N SM-3MG5O SM-3MG5P SM-3MG5Q SM-3MG5R SM-3MG5S SM-3MG5T
SM-3MG5U SM-3MG5V SM-3MG5W SM-3MG5Y SM-3MG5Z SM-3MG61 SM-3MG62 SM-3MG63 SM-3MG64 SM-3MG65 SM-3MG66
SM-3MG67 SM-3MG68 SM-3MG69 SM-3MG6A SM-3MG6B SM-3MGPL SM-3MGPN SM-3MGPO SM-3MGPP SM-3MGPQ SM-3MGPR
SM-3MGPS SM-3MGPU SM-3MGPV SM-3MGPW SM-3MGPX SM-3MGPY SM-3MGPZ
GC-content (%)
Read position (bp)

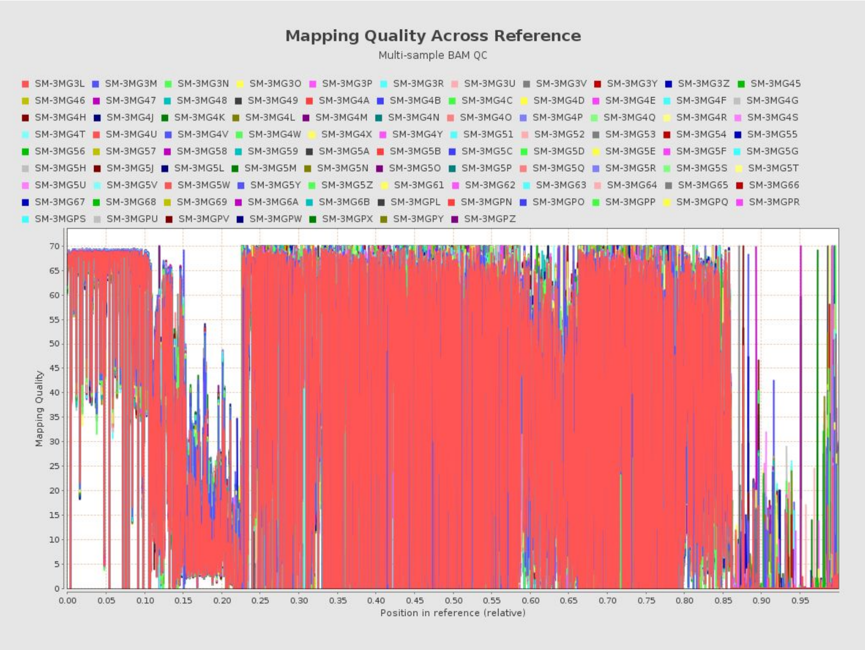
Mapping Quality Across Reference
Multi-sample BAM QC
SM-3MG3L SM-3MG3M SM-3MG3N SM-3MG3O SM-3MG3P SM-3MG3R SM-3MG3U SM-3MG3V SM-3MG3Y SM-3MG3Z SM-3MG45
SM-3MG46 SM-3MG47 SM-3MG48 SM-3MG49 SM-3MG4A SM-3MG4B SM-3MG4C SM-3MG4D SM-3MG4E SM-3MG4F SM-3MG4G
SM-3MG4H SM-3MG4J SM-3MG4K SM-3MG4L SM-3MG4M SM-3MG4N SM-3MG4O SM-3MG4P SM-3MG4Q SM-3MG4R SM-3MG4S
SM-3MG4T SM-3MG4U SM-3MG4V SM-3MG4W SM-3MG4X SM-3MG4Y SM-3MG51 SM-3MG52 SM-3MG53 SM-3MG54 SM-3MG55
SM-3MG56 SM-3MG57 SM-3MG58 SM-3MG59 SM-3MG5A SM-3MG5B SM-3MG5C SM-3MG5D SM-3MG5E SM-3MG5F SM-3MG5G
SM-3MG5H SM-3MG5J SM-3MG5L SM-3MG5M SM-3MG5N SM-3MG5O SM-3MG5P SM-3MG5Q SM-3MG5R SM-3MG5S SM-3MG5T
SM-3MG5U SM-3MG5V SM-3MG5W SM-3MG5Y SM-3MG5Z SM-3MG61 SM-3MG62 SM-3MG63 SM-3MG64 SM-3MG65 SM-3MG66
SM-3MG67 SM-3MG68 SM-3MG69 SM-3MG6A SM-3MG6B SM-3MGPL SM-3MGPN SM-3MGPO SM-3MGPP SM-3MGPQ SM-3MGPR
SM-3MGPS SM-3MGPU SM-3MGPV SM-3MGPW SM-3MGPX SM-3MGPY SM-3MGPZ
Mapping Quality
0 5 10 15 20 25 30 35 40 45 50 55 60 65 70
0.00 0.05 0.10 0.15 0.20 0.25 0.30 0.35 0.40 0.45 0.50 0.55 0.60 0.65 0.70 0.75 0.80 0.85 0.90 0.95
Position in reference (relative)

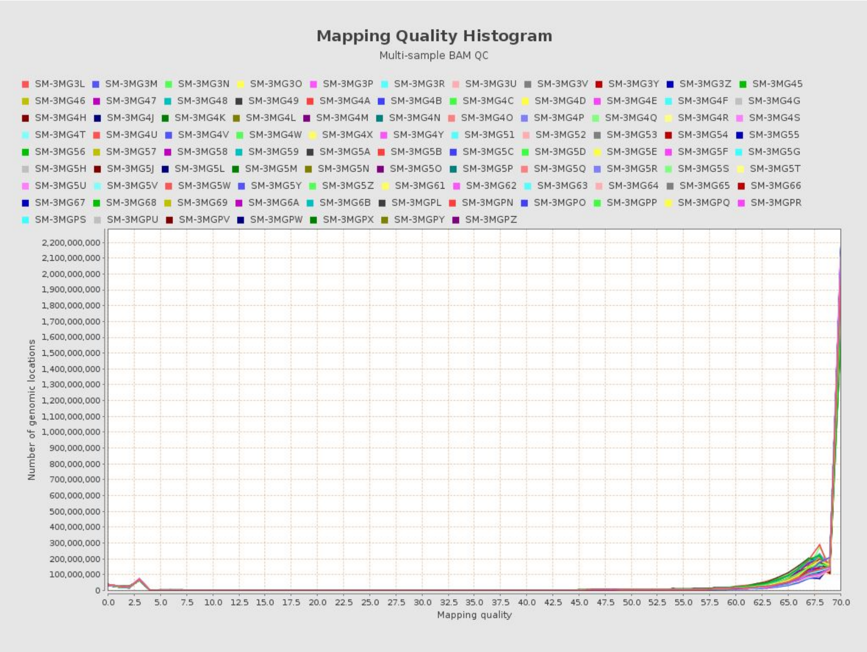
Mapping Quality Histogram
Multi-sample BAM QC
SM-3MG3L SM-3MG3M SM-3MG3N SM-3MG3O SM-3MG3P SM-3MG3R SM-3MG3U SM-3MG3V SM-3MG3Y SM-3MG3Z SM-3MG45
SM-3MG46 SM-3MG47 SM-3MG48 SM-3MG49 SM-3MG4A SM-3MG4B SM-3MG4C SM-3MG4D SM-3MG4E SM-3MG4F SM-3MG4G
SM-3MG4H SM-3MG4J SM-3MG4K SM-3MG4L SM-3MG4M SM-3MG4N SM-3MG4O SM-3MG4P SM-3MG4Q SM-3MG4R SM-3MG4S
SM-3MG4T SM-3MG4U SM-3MG4V SM-3MG4W SM-3MG4X SM-3MG4Y SM-3MG51 SM-3MG52 SM-3MG53 SM-3MG54 SM-3MG55
SM-3MG56 SM-3MG57 SM-3MG58 SM-3MG59 SM-3MG5A SM-3MG5B SM-3MG5C SM-3MG5D SM-3MG5E SM-3MG5F SM-3MG5G
SM-3MG5H SM-3MG5J SM-3MG5L SM-3MG5M SM-3MG5N SM-3MG5O SM-3MG5P SM-3MG5Q SM-3MG5R SM-3MG5S SM-3MG5T
SM-3MG5U SM-3MG5V SM-3MG5W SM-3MG5Y SM-3MG5Z SM-3MG61 SM-3MG62 SM-3MG63 SM-3MG64 SM-3MG65 SM-3MG66
SM-3MG67 SM-3MG68 SM-3MG69 SM-3MG6A SM-3MG6B SM-3MGPL SM-3MGPN SM-3MGPO SM-3MGPP SM-3MGPQ SM-3MGPR
SM-3MGPS SM-3MGPU SM-3MGPV SM-3MGPW SM-3MGPX SM-3MGPY SM-3MGPZ
Number of genomic locations
0 100,000,000 200,000,000 300,000,000 400,000,000 500,000,000 600,000,000 700,000,000 800,000,000 900,000,000 1,000,000,000 1,100,000,000 1,200,000,000 1,300,000,000 1,400,000,000 1,500,000,000 1,600,000,000 1,700,000,000 1,800,000,000 1,900,000,000 2,000,000,000 2,100,000,000 2,200,000,000
0.0 2.5 5.0 7.5 10.0 12.5 15.0 17.5 20.0 22.5 25.0 27.5 30.0 32.5 35.0 37.5 40.0 42.5 45.0 47.5 50.0 52.5 55.0 57.5 60.0 62.5 65.0 67.5 70.0
Mapping quality

SUPPORTING METHODS

Phylogenetic relationships. The pipeline for running treemix and f3 statistics can be downloaded: https://github.com/jbv2/nf-vcf2treemix. In brief, from the IPVS we selected the NM, NP, and 4 CHB individuals from the 1000 genomes project (samples ids: NA18639, NA18640, NA18641, NA18642). We kept biallelic SNVs with a MAF > 0.05 with bcftools, and removed variants in linkage disequilibrium (r2 > 0.85) with bcftools +prune plugin using parameters *--window 2000bp --nsites-per-win 1*. We ran TreeMix [4] with the parameters *-k 1000 -global -root CHB -bootstrap 100 -m 0 -noss -seed 99*. We then inferred one (*-m 1*) and two (*-m 2*) migration events using the previous tree topology as a guide with the -g parameter.

SUPPORTING REFERENCES

1. Karczewski KJ, Francioli LC, Tiao G, Cummings BB, Alföldi J, Wang Q, et al. The mutational constraint spectrum quantified from variation in 141,456 humans. bioRxiv. 2020. p. 531210. doi:10.1101/531210

2. Moreno-Estrada A, Gignoux CR, Fernández-López JC, Zakharia F, Sikora M, Contreras AV, et al. Human genetics. The genetics of Mexico recapitulates Native American substructure and affects biomedical traits. Science. 2014;344: 1280–1285.

3. Ávila-Arcos MC, McManus KF, Sandoval K, Rodríguez-Rodríguez JE, Villa-Islas V, Martin AR, et al. Population History and Gene Divergence in Native Mexicans Inferred from 76 Human Exomes. Mol Biol Evol. 2020;37: 994–1006.

4. Pickrell J, Pritchard J. Inference of population splits and mixtures from genome-wide allele frequency data. Nature Precedings. 2012. doi:10.1038/npre.2012.6956.1
