# Supplementary material for: Whole genome variation in 27 Mexican indigenous populations, demographic and biomedical insights
Source: PLoS One. 2021 Apr 8;16(4):e0249773. doi: 10.1371/journal.pone.0249773 (PMC8031408; doi:10.1371/journal.pone.0249773)
Supplement: S1 Data — (DOCX) [file pone.0249773.s003.docx]

**Data Access Request Form for the 100G-MX Project**

| **Contact Information** | |
| --- | --- |
| **Name** |  |
| **Email** |  |
| **Job title** |  |
| **Institution** |  |
| **Institutional address** |  |

Dear Garbiñe Saruwatari Zavala ([gsaruwatari@inmegen.gob.mx](mailto:gsaruwatari@inmegen.gob.mx)), President of the Ethics Committee at Instituto Nacional de Medicina Genómica (INMEGEN).

I request access to the following data from the 100G-MX project:

|  |
| --- |

With regards to the non-public whole genome individual data from the 100G-MX Project,

1. I will not use the data for any commercial purposes, I will use it only for scientific purposes;
2. I understand that the individuals included in this study are potentially vulnerable participants from underrepresented Native Mexican minorities;
3. I will make no attempt to connect the generic data to personal identifiers for the individuals;
4. I will not post the data publicly;
5. I will not distribute or make use of the data outside of the following stated research intentions:

|  |
| --- |

Sincerely,

| **Signature** |  |
| --- | --- |
| **Name** |  |
| **Date** |  |
