# Supplementary material for: Whole genome variation in 27 Mexican indigenous populations, demographic and biomedical insights
Source: PLoS One. 2021 Apr 8;16(4):e0249773. doi: 10.1371/journal.pone.0249773 (PMC8031408; doi:10.1371/journal.pone.0249773)

Qualimap Analysis Results

Multi-sample BAM QC analysis

Generated by Qualimap v.2.2.1

2017/07/31 16:49:22

1. Input data & parameters

1.1. Samples

SM-3MG6B	/100g/analysis/004/results/wholeGenome/SM-3MG6B.markdup_stats
SM-3MG6A	/100g/analysis/004/results/wholeGenome/SM-3MG6A.markdup_stats
SM-3MG5Y	/100g/analysis/004/results/wholeGenome/SM-3MG5Y.markdup_stats
SM-3MG5Z	/100g/analysis/004/results/wholeGenome/SM-3MG5Z.markdup_stats
SM-3MG5W	/100g/analysis/004/results/wholeGenome/SM-3MG5W.markdup_stats
SM-3MG68	/100g/analysis/004/results/wholeGenome/SM-3MG68.markdup_stats
SM-3MG69	/100g/analysis/004/results/wholeGenome/SM-3MG69.markdup_stats
SM-3MG5U	/100g/analysis/004/results/wholeGenome/SM-3MG5U.markdup_stats
SM-3MG66	/100g/analysis/004/results/wholeGenome/SM-3MG66.markdup_stats
SM-3MG5V	/100g/analysis/004/results/wholeGenome/SM-3MG5V.markdup_stats
SM-3MG67	/100g/analysis/004/results/wholeGenome/SM-3MG67.markdup_stats
SM-3MG5S	/100g/analysis/004/results/wholeGenome/SM-3MG5S.markdup_stats
SM-3MG64	/100g/analysis/004/results/wholeGenome/SM-3MG64.markdup_stats

SM-3MG5T	/100g/analysis/004/results/wholeGenome/SM-3MG5T.markdup_stats
SM-3MG65	/100g/analysis/004/results/wholeGenome/SM-3MG65.markdup_stats
SM-3MG5Q	/100g/analysis/004/results/wholeGenome/SM-3MG5Q.markdup_stats
SM-3MG62	/100g/analysis/004/results/wholeGenome/SM-3MG62.markdup_stats
SM-3MG5R	/100g/analysis/004/results/wholeGenome/SM-3MG5R.markdup_stats
SM-3MG63	/100g/analysis/004/results/wholeGenome/SM-3MG63.markdup_stats
SM-3MG5O	/100g/analysis/004/results/wholeGenome/SM-3MG5O.markdup_stats
SM-3MG5P	/100g/analysis/004/results/wholeGenome/SM-3MG5P.markdup_stats
SM-3MG61	/100g/analysis/004/results/wholeGenome/SM-3MG61.markdup_stats
SM-3MG5M	/100g/analysis/004/results/wholeGenome/SM-3MG5M.markdup_stats
SM-3MG5N	/100g/analysis/004/results/wholeGenome/SM-3MG5N.markdup_stats
SM-3MG5L	/100g/analysis/004/results/wholeGenome/SM-3MG5L.markdup_stats
SM-3MG5J	/100g/analysis/004/results/wholeGenome/SM-3MG5J.markdup_stats
SM-3MG5G	/100g/analysis/004/results/wholeGenome/SM-3MG5G.markdup_stats
SM-3MG5H	/100g/analysis/004/results/wholeGenome/SM-3MG5H.markdup_stats

SM-3MG5E	/100g/analysis/004/results/wholeGenome/SM-3MG5E.markdup_stats
SM-3MG5F	/100g/analysis/004/results/wholeGenome/SM-3MG5F.markdup_stats
SM-3MG5C	/100g/analysis/004/results/wholeGenome/SM-3MG5C.markdup_stats
SM-3MG5D	/100g/analysis/004/results/wholeGenome/SM-3MG5D.markdup_stats
SM-3MG5A	/100g/analysis/004/results/wholeGenome/SM-3MG5A.markdup_stats
SM-3MG5B	/100g/analysis/004/results/wholeGenome/SM-3MG5B.markdup_stats
SM-3MG4X	/100g/analysis/004/results/wholeGenome/SM-3MG4X.markdup_stats
SM-3MG59	/100g/analysis/004/results/wholeGenome/SM-3MG59.markdup_stats
SM-3MG4Y	/100g/analysis/004/results/wholeGenome/SM-3MG4Y.markdup_stats
SM-3MG4V	/100g/analysis/004/results/wholeGenome/SM-3MG4V.markdup_stats
SM-3MG57	/100g/analysis/004/results/wholeGenome/SM-3MG57.markdup_stats
SM-3MG4W	/100g/analysis/004/results/wholeGenome/SM-3MG4W.markdup_stats
SM-3MG58	/100g/analysis/004/results/wholeGenome/SM-3MG58.markdup_stats
SM-3MG4T	/100g/analysis/004/results/wholeGenome/SM-3MG4T.markdup_stats
SM-3MG55	/100g/analysis/004/results/wholeGenome/SM-3MG55.markdup_stats

SM-3MG4U	/100g/analysis/004/results/wholeGenome/SM-3MG4U.markdup_stats
SM-3MG56	/100g/analysis/004/results/wholeGenome/SM-3MG56.markdup_stats
SM-3MG4R	/100g/analysis/004/results/wholeGenome/SM-3MG4R.markdup_stats
SM-3MG53	/100g/analysis/004/results/wholeGenome/SM-3MG53.markdup_stats
SM-3MG4S	/100g/analysis/004/results/wholeGenome/SM-3MG4S.markdup_stats
SM-3MG54	/100g/analysis/004/results/wholeGenome/SM-3MG54.markdup_stats
SM-3MG4P	/100g/analysis/004/results/wholeGenome/SM-3MG4P.markdup_stats
SM-3MG51	/100g/analysis/004/results/wholeGenome/SM-3MG51.markdup_stats
SM-3MG4Q	/100g/analysis/004/results/wholeGenome/SM-3MG4Q.markdup_stats
SM-3MG52	/100g/analysis/004/results/wholeGenome/SM-3MG52.markdup_stats
SM-3MG4N	/100g/analysis/004/results/wholeGenome/SM-3MG4N.markdup_stats
SM-3MG4O	/100g/analysis/004/results/wholeGenome/SM-3MG4O.markdup_stats
SM-3MG4L	/100g/analysis/004/results/wholeGenome/SM-3MG4L.markdup_stats
SM-3MG4M	/100g/analysis/004/results/wholeGenome/SM-3MG4M.markdup_stats
SM-3MG4J	/100g/analysis/004/results/wholeGenome/SM-3MG4J.markdup_stats

SM-3MG4K	/100g/analysis/004/results/wholeGenome/SM-3MG4K.markdup_stats
SM-3MG4H	/100g/analysis/004/results/wholeGenome/SM-3MG4H.markdup_stats
SM-3MG4F	/100g/analysis/004/results/wholeGenome/SM-3MG4F.markdup_stats
SM-3MG4G	/100g/analysis/004/results/wholeGenome/SM-3MG4G.markdup_stats
SM-3MG4D	/100g/analysis/004/results/wholeGenome/SM-3MG4D.markdup_stats
SM-3MG4E	/100g/analysis/004/results/wholeGenome/SM-3MG4E.markdup_stats
SM-3MG4B	/100g/analysis/004/results/wholeGenome/SM-3MG4B.markdup_stats
SM-3MG4C	/100g/analysis/004/results/wholeGenome/SM-3MG4C.markdup_stats
SM-3MG4A	/100g/analysis/004/results/wholeGenome/SM-3MG4A.markdup_stats
SM-3MGPZ	/100g/analysis/004/results/wholeGenome/SM-3MGPZ.markdup_stats
SM-3MGPX	/100g/analysis/004/results/wholeGenome/SM-3MGPX.markdup_stats
SM-3MGPY	/100g/analysis/004/results/wholeGenome/SM-3MGPY.markdup_stats
SM-3MG3Y	/100g/analysis/004/results/wholeGenome/SM-3MG3Y.markdup_stats
SM-3MGPV	/100g/analysis/004/results/wholeGenome/SM-3MGPV.markdup_stats
SM-3MG3Z	/100g/analysis/004/results/wholeGenome/SM-3MG3Z.markdup_stats

SM-3MGPW	/100g/analysis/004/results/wholeGenome/SM-3MGPW.markdup_stats
SM-3MG48	/100g/analysis/004/results/wholeGenome/SM-3MG48.markdup_stats
SM-3MG49	/100g/analysis/004/results/wholeGenome/SM-3MG49.markdup_stats
SM-3MGPU	/100g/analysis/004/results/wholeGenome/SM-3MGPU.markdup_stats
SM-3MG3U	/100g/analysis/004/results/wholeGenome/SM-3MG3U.markdup_stats
SM-3MG46	/100g/analysis/004/results/wholeGenome/SM-3MG46.markdup_stats
SM-3MGPR	/100g/analysis/004/results/wholeGenome/SM-3MGPR.markdup_stats
SM-3MG3V	/100g/analysis/004/results/wholeGenome/SM-3MG3V.markdup_stats
SM-3MG47	/100g/analysis/004/results/wholeGenome/SM-3MG47.markdup_stats
SM-3MGPS	/100g/analysis/004/results/wholeGenome/SM-3MGPS.markdup_stats
SM-3MGPP	/100g/analysis/004/results/wholeGenome/SM-3MGPP.markdup_stats
SM-3MG45	/100g/analysis/004/results/wholeGenome/SM-3MG45.markdup_stats
SM-3MGPQ	/100g/analysis/004/results/wholeGenome/SM-3MGPQ.markdup_stats
SM-3MGPN	/100g/analysis/004/results/wholeGenome/SM-3MGPN.markdup_stats
SM-3MG3R	/100g/analysis/004/results/wholeGenome/SM-3MG3R.markdup_stats

SM-3MGPO	/100g/analysis/004/results/wholeGenome/SM-3MGPO.markdup_stats
SM-3MG3O	/100g/analysis/004/results/wholeGenome/SM-3MG3O.markdup_stats
SM-3MGPL	/100g/analysis/004/results/wholeGenome/SM-3MGPL.markdup_stats
SM-3MG3P	/100g/analysis/004/results/wholeGenome/SM-3MG3P.markdup_stats
SM-3MG3M	/100g/analysis/004/results/wholeGenome/SM-3MG3M.markdup_stats
SM-3MG3N	/100g/analysis/004/results/wholeGenome/SM-3MG3N.markdup_stats
SM-3MG3L	/100g/analysis/004/results/wholeGenome/SM-3MG3L.markdup_stats

2. Summary

2.1. Globals

Number of samples	95
Total number of mapped reads	50,726,214,735
Mean samples coverage	22.32
Mean samples GC-content	42.7
Mean samples mapping quality	32.35
Mean samples insert size	322.46

2.2. Sample statistics

**Sample name**	**Coverage mean**	**Coverage std**	**GC percentage**	**Mapping quality mean**	**Insert size median**
SM-3MG3L	21.6587	152.9206	43.63	32.2233	328.0
SM-3MG3M	29.2731	194.9304	43.83	32.5356	330.0
SM-3MG3N	22.7656	159.3852	42.9	31.9319	325.0
SM-3MG3O	23.9115	219.0725	43.13	32.3938	328.0
SM-3MG3P	19.0812	123.2319	42.71	32.34	336.0
SM-3MG3R	21.1342	119.143	42.69	32.382	317.0
SM-3MG3U	17.4147	98.7744	42.85	31.4635	329.0
SM-	18.513	113.1231	42.74	31.9379	338.0

3MG3V					
SM-3MG3Y	17.9934	112.0233	43.13	32.8015	328.0
SM-3MG3Z	17.7478	137.398	43.19	31.4151	341.0
SM-3MG45	20.3735	136.4201	41.66	31.7285	352.0
SM-3MG46	22.9783	152.3342	41.26	32.4826	333.0
SM-3MG47	20.2045	114.4378	41.54	32.2962	352.0
SM-3MG48	22.8778	110.9619	41.68	31.997	322.0
SM-3MG49	18.7559	92.3984	41.75	32.3334	354.0
SM-3MG4A	22.2546	133.3291	41.08	30.8161	365.0
SM-3MG4B	22.0681	128.2228	42.16	32.9558	324.0
SM-3MG4C	18.151	121.0866	41.7	32.2363	345.0
SM-3MG4D	22.2237	110.3735	41.25	31.8661	312.0
SM-3MG4E	19.0716	128.6994	42.32	30.772	304.0
SM-3MG4F	22.8857	156.7791	43.16	32.5706	304.0
SM-3MG4G	18.8903	97.8404	42.42	31.4414	314.0
SM-3MG4H	20.8813	109.9448	42.64	32.2824	315.0
SM-3MG4J	23.4749	186.249	44.04	32.6646	320.0
SM-3MG4K	24.0074	181.5293	43.51	32.6862	328.0

SM-3MG4L	19.5513	125.189	43.63	31.9536	324.0
SM-3MG4M	22.1197	157.1556	44.57	31.6728	325.0
SM-3MG4N	23.7962	135.6849	44.66	32.6768	329.0
SM-3MG4O	26.4489	155.7175	44.75	32.9359	319.0
SM-3MG4P	24.0564	146.0307	44.09	32.5389	342.0
SM-3MG4Q	18.7233	131.0039	44.93	31.7394	342.0
SM-3MG4R	20.0608	99.6618	43.9	31.6552	331.0
SM-3MG4S	21.4691	137.1144	44.26	32.202	338.0
SM-3MG4T	19.3468	120.6925	44.2	32.3027	327.0
SM-3MG4U	21.9997	122.3196	44.58	32.2057	328.0
SM-3MG4V	20.6735	140.594	44.64	32.6744	338.0
SM-3MG4W	24.1261	130.7845	44.19	32.4064	331.0
SM-3MG4X	23.6906	132.7424	44.21	32.3124	332.0
SM-3MG4Y	22.3387	163.9001	44.25	32.6203	334.0
SM-3MG51	22.5792	129.4243	43.76	33.1825	315.0
SM-3MG52	22.9021	131.9716	43.17	32.8003	297.0

SM-3MG53	22.3951	121.3921	42.5	32.3213	336.0
SM-3MG54	23.679	141.6705	43.49	32.3241	322.0
SM-3MG55	19.5004	119.0294	42.26	32.4886	306.0
SM-3MG56	22.2535	102.4978	41.44	34.1148	317.0
SM-3MG57	21.9237	116.3828	41.9	32.8717	321.0
SM-3MG58	25.2753	150.8868	42.05	32.9061	315.0
SM-3MG59	24.9409	97.5464	42.64	32.4095	308.0
SM-3MG5A	27.4988	147.096	42.58	32.9982	323.0
SM-3MG5B	26.1932	144.4061	42.5	32.6209	303.0
SM-3MG5C	19.9535	100.1962	42.5	33.2327	314.0
SM-3MG5D	20.2284	112.1076	42.82	31.8381	307.0
SM-3MG5E	21.439	91.4881	41.94	34.1078	318.0
SM-3MG5F	24.1018	127.3575	41.94	32.8897	302.0
SM-3MG5G	26.9722	192.79	42.0	32.6228	303.0
SM-3MG5H	27.1039	151.6742	41.88	31.8476	299.0
SM-3MG5J	29.324	148.8433	42.68	32.763	307.0
SM-3MG5L	27.5186	130.0132	42.25	32.8991	308.0
SM-3MG5M	27.333	217.358	43.47	32.6401	298.0
SM-	22.9432	118.7197	42.62	32.8106	309.0

3MG5N					
SM-3MG5O	28.0944	148.605	42.7	32.7211	298.0
SM-3MG5P	23.2966	134.6972	42.53	32.8505	315.0
SM-3MG5Q	26.9745	117.455	41.75	32.7806	309.0
SM-3MG5R	23.9235	112.8134	40.95	32.1513	323.0
SM-3MG5S	27.7617	137.1195	41.7	32.101	327.0
SM-3MG5T	19.082	93.9677	40.97	31.8197	311.0
SM-3MG5U	20.424	98.6234	41.68	32.5787	332.0
SM-3MG5V	21.4199	125.9751	40.79	31.8922	332.0
SM-3MG5W	22.1361	123.3379	41.76	32.7256	344.0
SM-3MG5Y	19.7806	100.2532	42.13	32.3001	343.0
SM-3MG5Z	18.9527	87.1285	42.07	32.2927	343.0
SM-3MG61	23.7512	114.6967	42.06	32.7774	323.0
SM-3MG62	26.2984	137.7507	42.94	31.9611	329.0
SM-3MG63	23.3631	153.4228	41.21	32.8453	338.0
SM-3MG64	23.2222	130.4554	40.82	31.9148	338.0
SM-3MG65	22.41	111.5035	42.23	32.0589	329.0
SM-3MG66	22.4212	133.0557	42.96	32.2821	332.0

SM-3MG67	17.941	117.2279	41.66	32.4631	337.0
SM-3MG68	19.5989	128.101	43.91	32.285	318.0
SM-3MG69	19.9151	158.6328	42.64	32.6379	319.0
SM-3MG6A	25.1824	138.6221	44.05	31.8977	323.0
SM-3MG6B	22.3182	152.1607	43.8	31.7881	314.0
SM-3MGPL	23.2325	139.4089	44.07	32.032	311.0
SM-3MGPN	22.2256	136.2239	41.9	31.685	307.0
SM-3MGPO	21.169	132.0733	43.94	32.1247	311.0
SM-3MGPP	19.5317	164.9409	43.01	32.2547	299.0
SM-3MGPQ	23.6949	121.7769	43.65	32.1764	307.0
SM-3MGPR	18.3693	107.5477	43.14	31.5727	329.0
SM-3MGPS	23.1041	122.5027	41.57	32.2952	307.0
SM-3MGPU	17.8242	135.7951	42.26	31.0712	318.0
SM-3MGPV	22.8302	144.741	42.93	33.6465	304.0
SM-3MGPW	20.4148	96.2905	40.75	32.5759	317.0
SM-3MGPX	23.4395	136.5546	42.05	32.5713	307.0

SM-3MGPY	21.0242	128.0753	41.61	32.2711	315.0
SM-3MGPZ	22.4502	142.4084	42.16	32.5874	313.0

3. Results : PCA

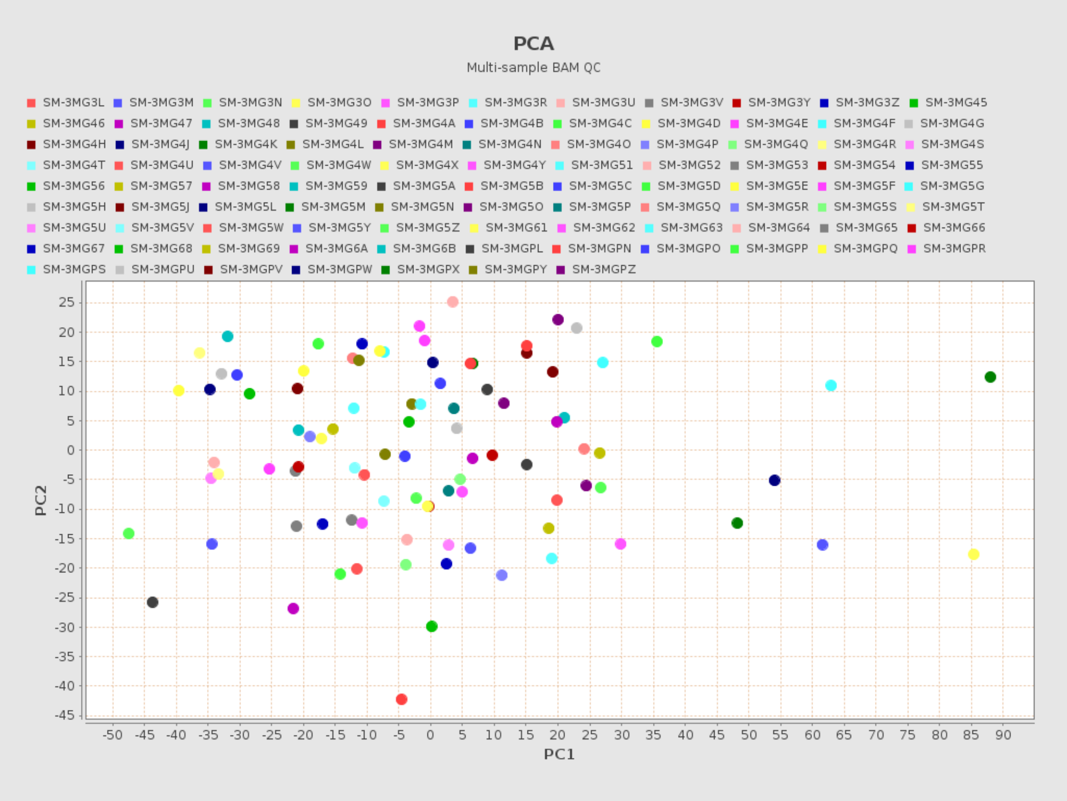

4. Results : Coverage Across Reference

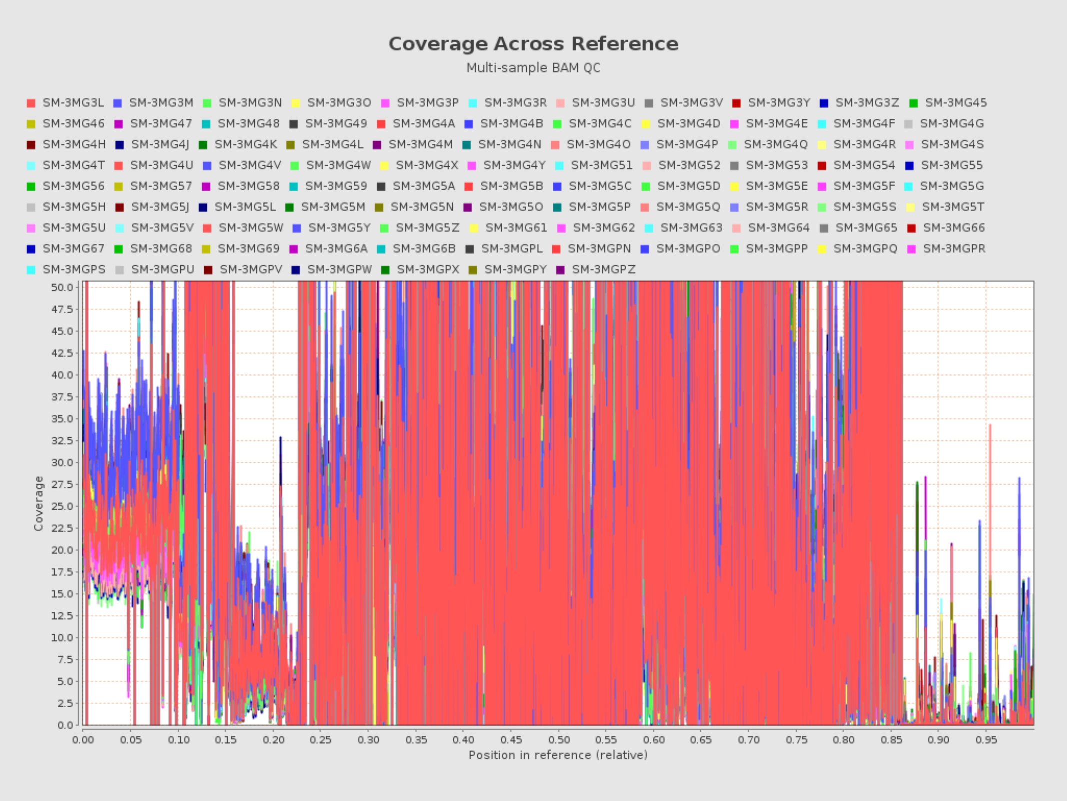

5. Results : Coverage Histogram (0 - 412436X)

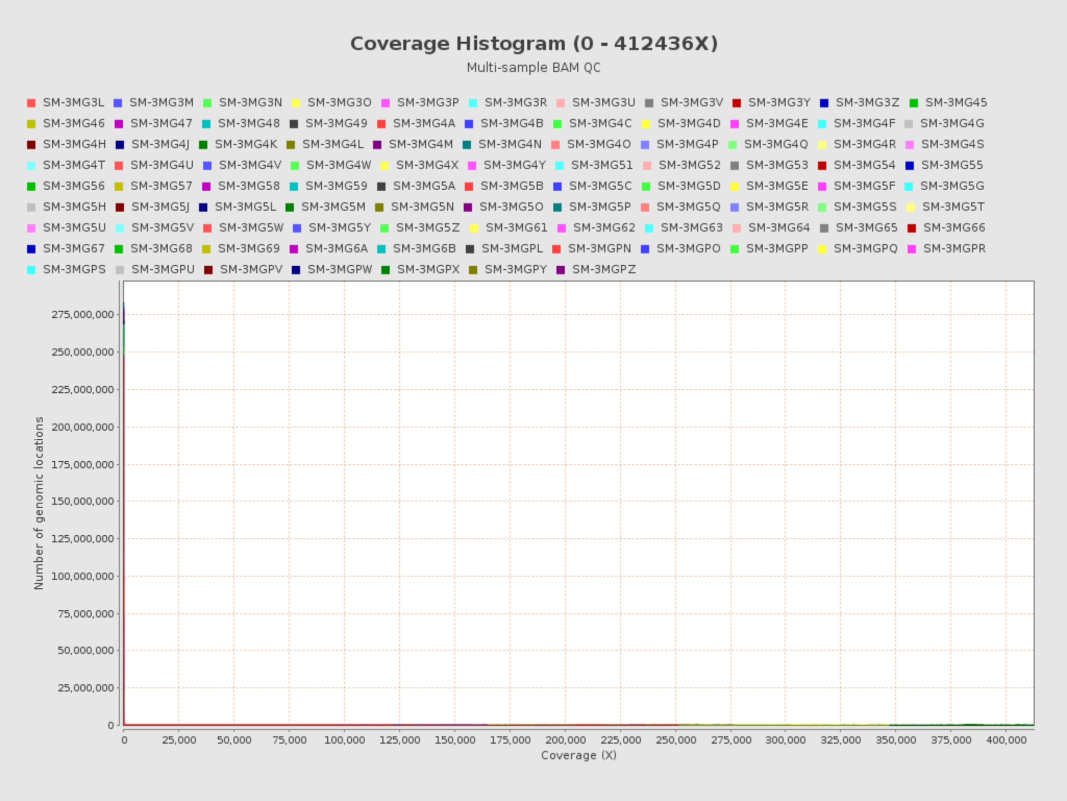

6. Results : Global Coverage Histogram

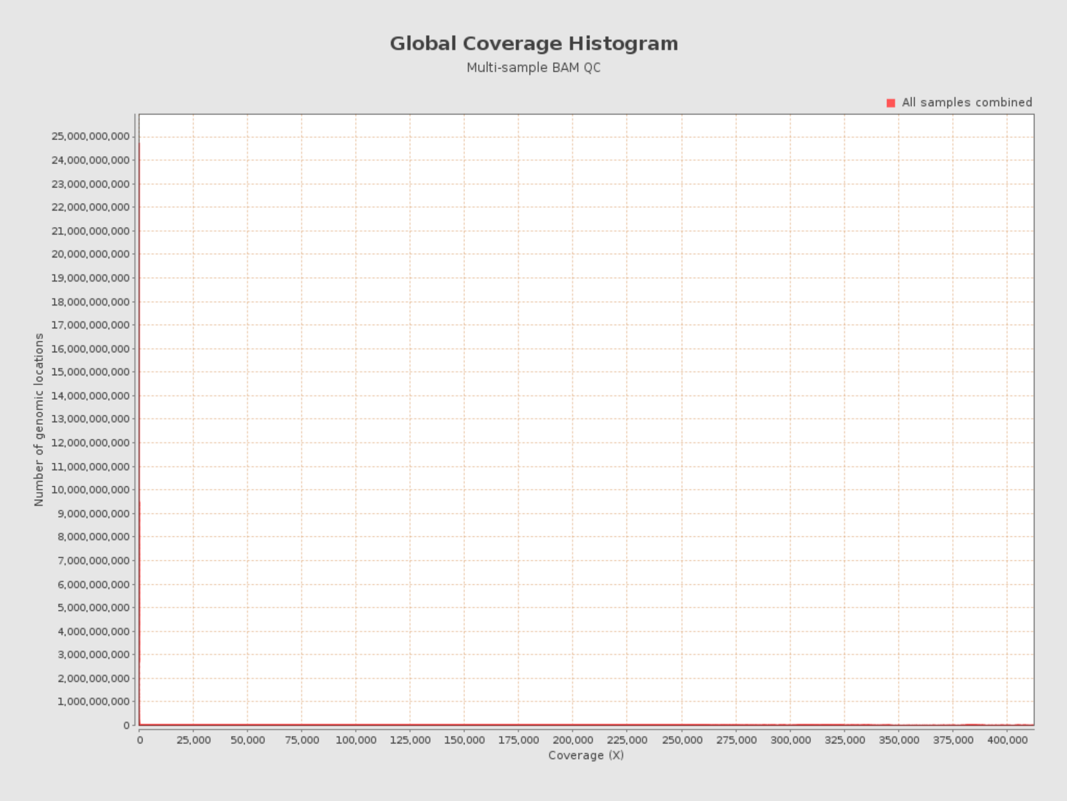

7. Results : Genome Fraction Coverage

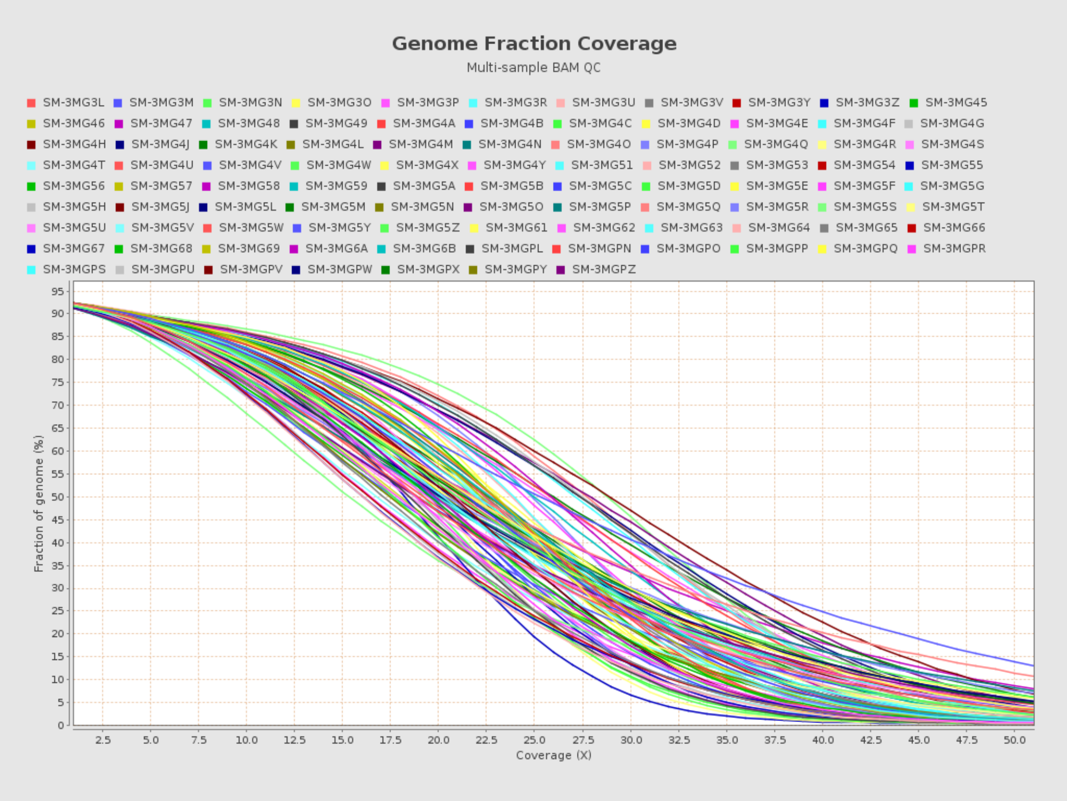

8. Results : Duplication Rate Histogram

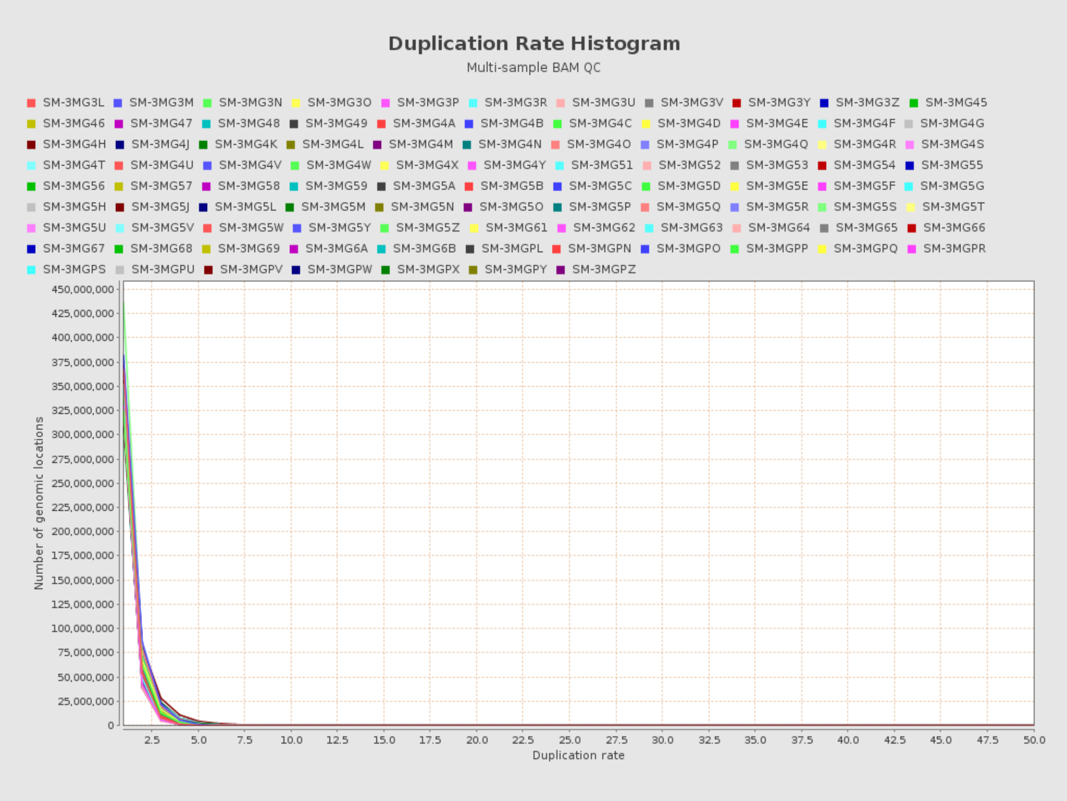

9. Results : Mapped reads GC-content

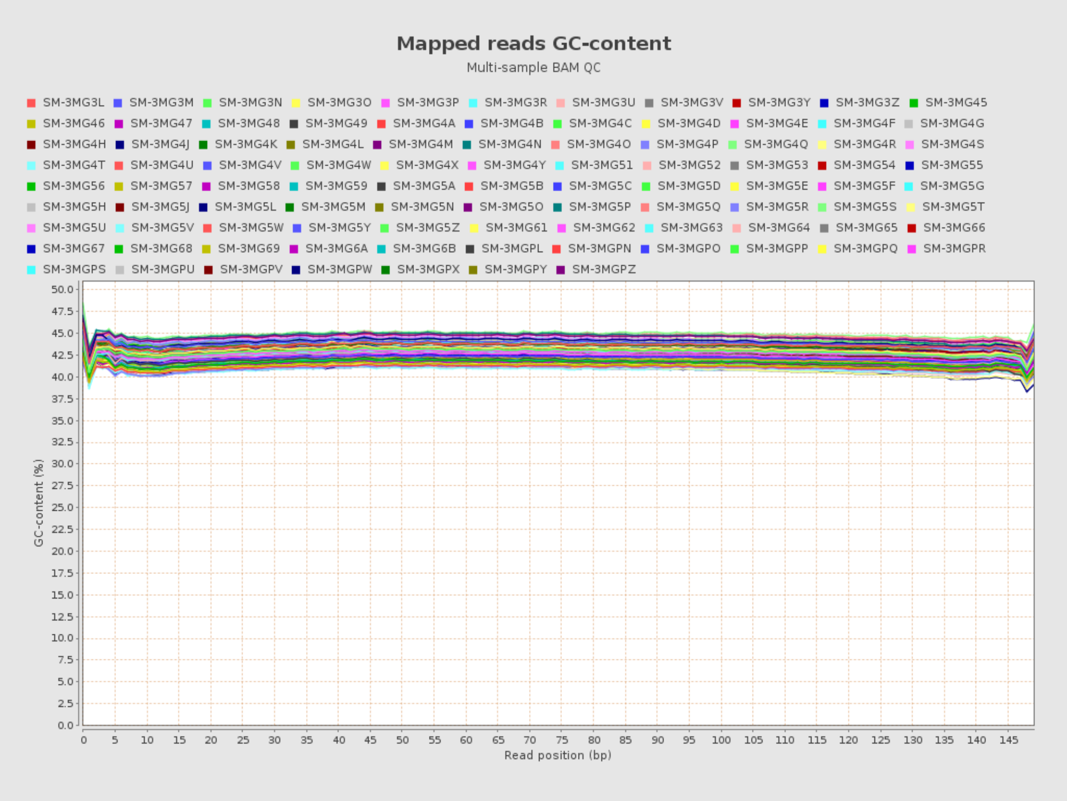

10. Results : Mapped Reads Clipping Profile

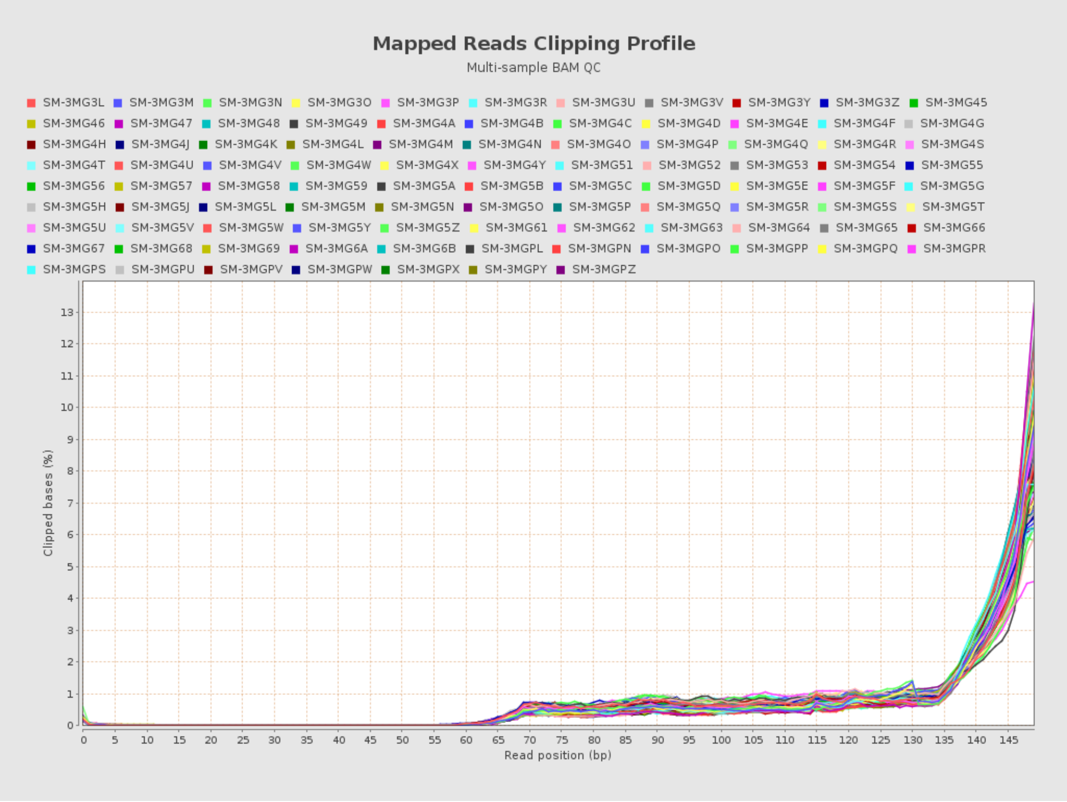

11. Results : Mapped Reads GC-content Distribution

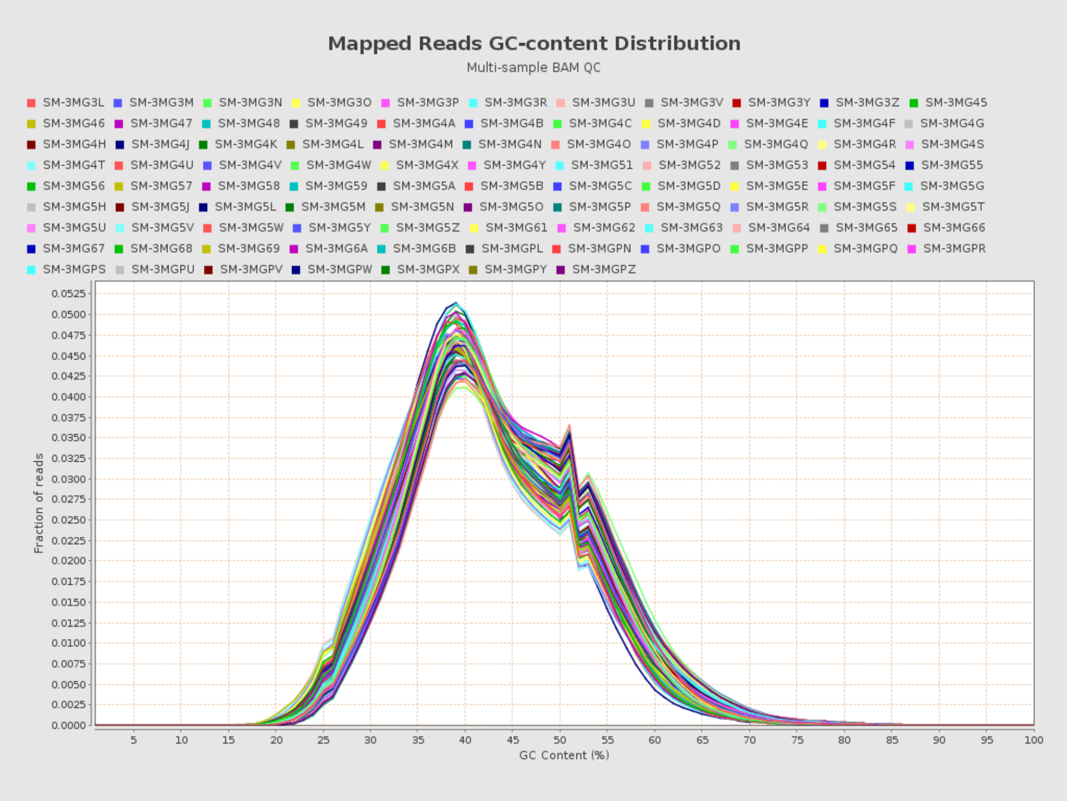

12. Results : Mapping Quality Across Reference

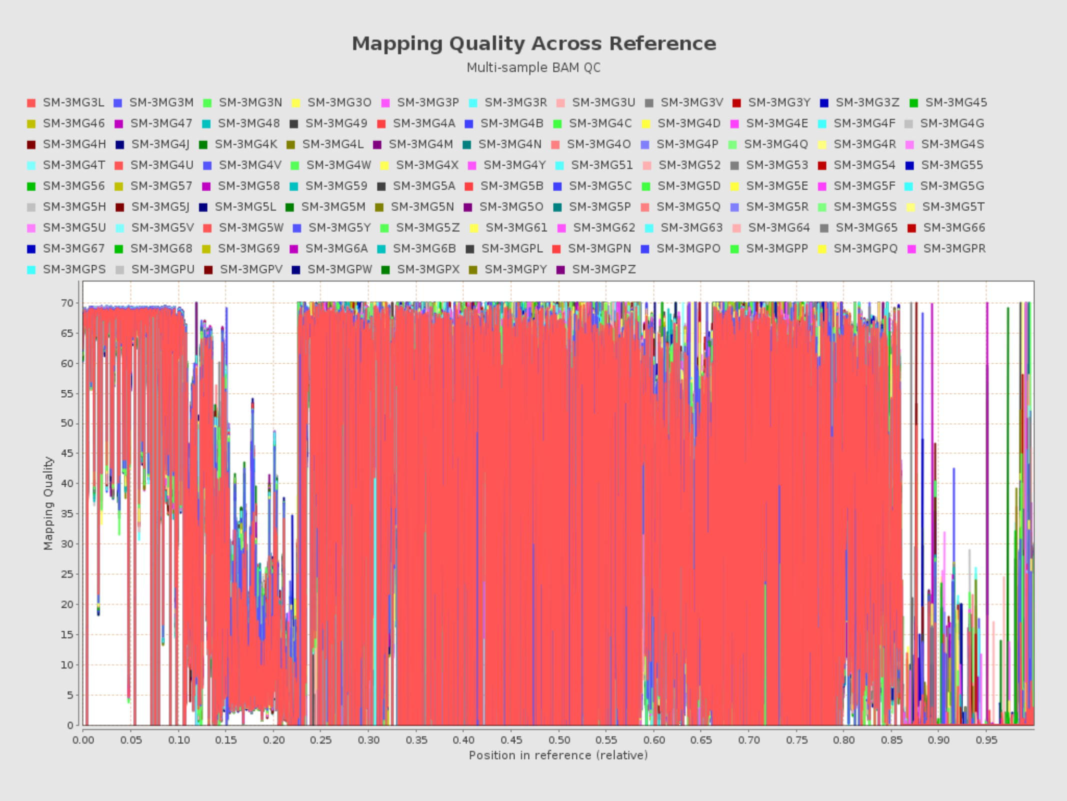

13. Results : Mapping Quality Histogram

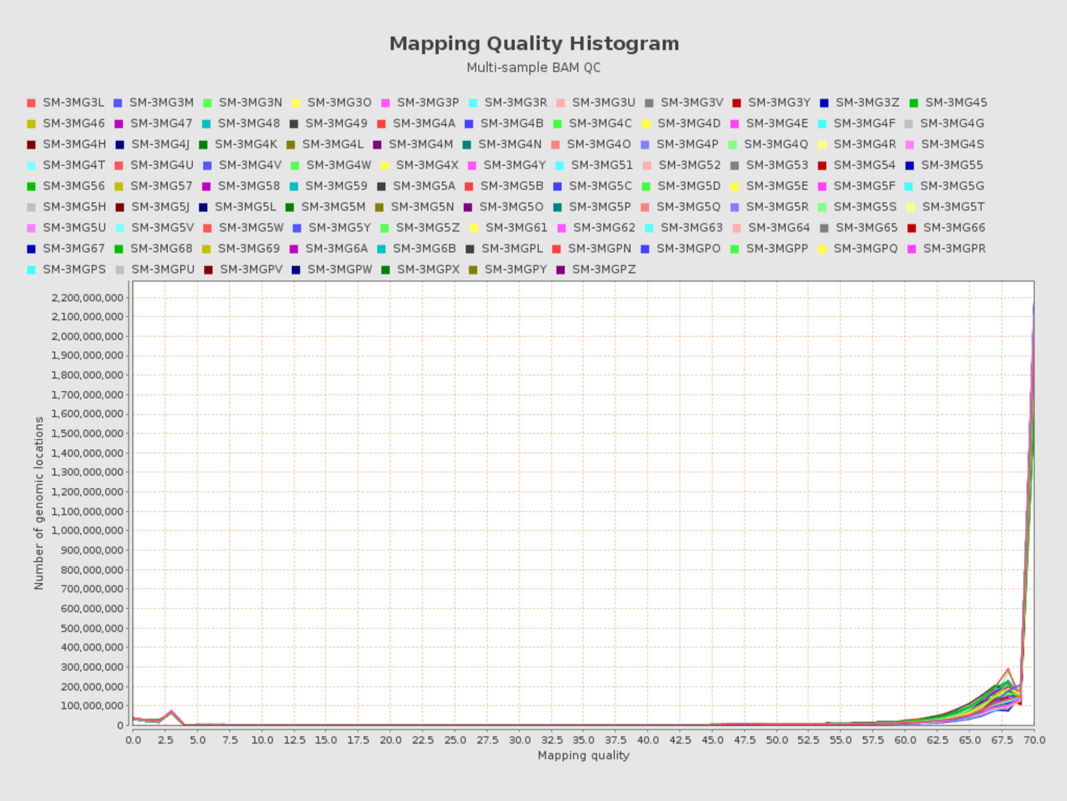

14. Results : Insert Size Across Reference

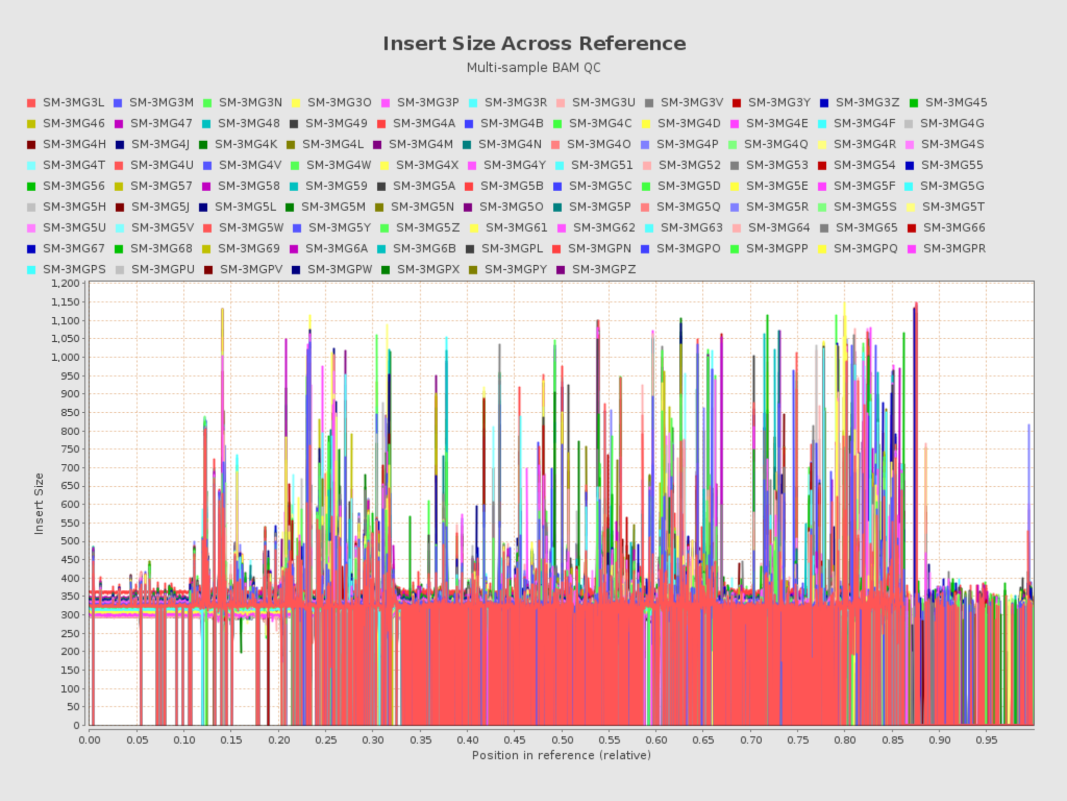

15. Results : Insert Size Histogram

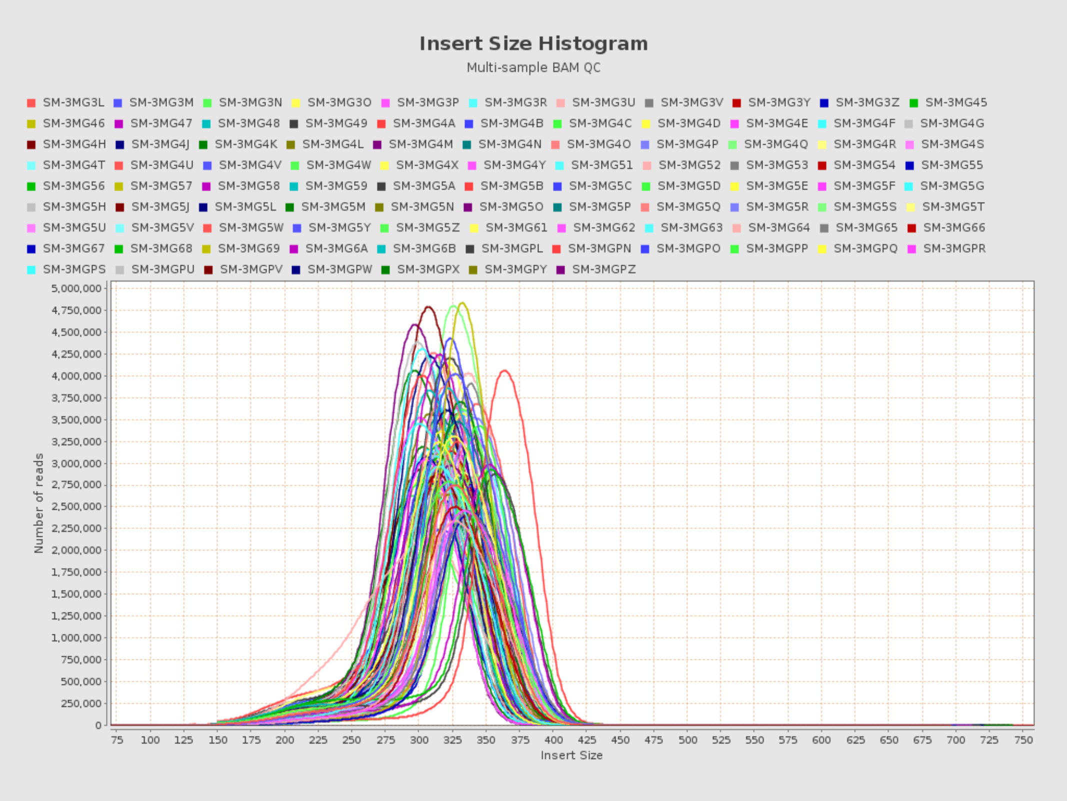

Supplement: S4 Data — (ZIP) [file pone.0249773.s006.zip › 100gmx_Qualimap_report.pdf]
